# Brown Seaweeds for the Management of Metabolic Syndrome and Associated Diseases

**DOI:** 10.3390/molecules25184182

**Published:** 2020-09-12

**Authors:** Daniela Gabbia, Sara De Martin

**Affiliations:** Department of Pharmaceutical and Pharmacological Sciences, University of Padova, 35131 Padova, Italy

**Keywords:** seaweeds, *Undaria pinnatifida*, *Ascophyllum nodosum*, *Laminaria japonica*, *Fucus vesiculosus*, metabolic syndrome, cardiovascular diseases, hypertension, obesity, NAFLD, diabetes

## Abstract

Metabolic syndrome is characterized by the coexistence of different metabolic disorders which increase the risk of developing type 2 diabetes mellitus and cardiovascular diseases. Therefore, metabolic syndrome leads to a reduction in patients’ quality of life as well as to an increase in morbidity and mortality. In the last few decades, it has been demonstrated that seaweeds exert multiple beneficial effects by virtue of their micro- and macronutrient content, which could help in the management of cardiovascular and metabolic diseases. This review aims to provide an updated overview on the potential of brown seaweeds for the prevention and management of metabolic syndrome and its associated diseases, based on the most recent evidence obtained from in vitro and in vivo preclinical and clinical studies. Owing to their great potential for health benefits, brown seaweeds are successfully used in some nutraceuticals and functional foods for treating metabolic syndrome comorbidities. However, some issues still need to be tackled and deepened to improve the knowledge of their ADME/Tox profile in humans, in particular by finding validated indexes of their absorption and obtaining reliable information on their efficacy and long-term safety.

## 1. Introduction

Metabolic syndrome (MS) is a cluster of different metabolic disorders—for example, obesity, hypertriglyceridemia, dyslipidemia, hyperglycemia, insulin resistance, hypertension, and pro-inflammatory and pro-thrombotic states. Together, these conditions lead to an increased risk of developing type 2 diabetes mellitus (T2DM) and atherosclerotic cardiovascular diseases (CVDs), thereby reducing patients’ quality of life and increasing morbidity and mortality [1]. As far as CVDs are concerned, according to the World Health Organization (WHO), CVD-related mortality is rapidly increasing in the world, since 17.5 million deaths occurred in 2012, and these are estimated to reach 22.2 million in 2030 [2]. In Western countries, the increased prevalence of CVDs and atherosclerosis, which actually accounted for approximately 50% of all CVD-related deaths, is further sustained by a sedentary lifestyle and high-calorie food intake [3]. It has been estimated that, in 2016, more than 1.9 billion adults (>18 of age) were overweight and 650 million were obese. A diet rich in fat and sugar and a lack of exercise cause an imbalance of energy status, leading to the accumulation of visceral fat, the development of liver steatosis, and the onset of MS risk factors. Since the prevalence of all these metabolic dysfunctions increased worldwide in the last years, it is essential to find new strategies for preventing or treating obesity, dyslipidemia, and insulin resistance [3], which are all well-recognized risk factors for MS.

The first-line therapy for MS involves drugs treating MS comorbidities and their symptoms. Although these drugs can be helpful, many of them lead to serious side-effects, and their efficacy could be reduced or lost with chronic administration. Thus, nutritional interventions have been proposed to reduce the risk of MS by preventing and alleviating chronic dietary-associated disorders [4,5].

In the last few decades, it has been demonstrated that a number of natural compounds exert multiple beneficial effects, such as anti-inflammatory, anti-hyperglycemic, and lipid-lowering activities, ameliorating blood lipid and glucose levels and insulin sensitivity [3,6,7]. Among these natural products, growing interest has been given to seaweeds, by virtue of their micro- and macronutrient content. They have been and are currently used in nutraceuticals and functional foods for the management of MS comorbidities [8]. Further supporting the effectiveness of seaweeds in treating and preventing MS, much epidemiological evidence has demonstrated that a lower incidence of obesity and diet-related disease is reported in countries where seaweeds are regularly consumed within the diet (for example, Japan) with respect to Western countries [9,10,11].

Seaweeds display peculiar chemical properties compared to terrestrial plants by virtue of their mineral-rich marine habitat, which requires specific adaptative responses for their survival. Thus, seaweeds are able to generate many bioactive metabolites with antioxidant and antimicrobial properties to counteract the abiotic stress typical of the marine environment—e.g., UV photo-damage, high salinity, constant oxygen exposure, and biotic stress induced by bacterial colonization and marine herbivores [8]. Interestingly, these bioactive compounds can be easily extracted and purified by novel eco-friendly techniques [12,13,14].

Seaweeds were classified into three main classes or phyla: brown seaweeds (*Ochrophyta)*, red seaweeds (*Rhodophyta*), and green seaweeds (*Chlorophyta*). Each phylum comprises thousands of algal species, many of which have been used since old times as food, folk remedies, dyes, and fertilizers [8,15,16]. Among the traditional dishes of East Asian countries (Korea, China, and Japan), many brown seaweeds are used—for example, in Wakame (*Undaria*), Konbu (*Laminaria*), Nori/Gim, and Hijiki (*Hizikia*). They represent a high-quality and healthy ingredient for food preparation by virtue of their low content of lipids and high content of polysaccharides, fibers, and polyunsaturated fatty acids (PUFAs). Moreover, they are rich in vitamins; minerals; and bioactive secondary metabolites—e.g., polyphenols, fucoidans, pigments, mycosporine-like amino acids (MAAs), and terpenoids [17,18].

This review aims to provide an updated overview of the potential of brown seaweeds for the prevention and management of MS and associated diseases, based on the most recent evidence obtained from in vitro and in vivo preclinical and clinical studies.

## 2. Metabolic Syndrome Mechanisms and Comorbidities

MS was described for the first time in the 1920s by a Swedish physician, who defined it as a cluster of hypertension, hyperglycemia, and gout. In 1940s, Vague observed that upper body adiposity was commonly associated with metabolic dysfunctions, T2DM, and CVD. These metabolic dysfunctions included glucose intolerance, T2DM, insulin resistance, central obesity, dyslipidemia, and hypertension, and were associated with an increased risk of CVDs [1]. Several parameters were proposed for the diagnosis of MS, generally including atherogenic dyslipidemia, elevated blood pressure, hyperglycemia, a prothrombotic and proinflammatory state, increased waist circumference, and/or obesity.

Apart from the aforementioned risk factors, the development of MS, a condition which is still widely present and increasing over time, is influenced by familial history and environmental and epigenetic conditions, such as a sedentary lifestyle and excessive intake of high-calorie foods [19]. Since the effective treatment of all these underlying risk factors could efficiently ameliorate MS and its related CVD risk, there is an urgent need for novel and effective strategies to prevent these comorbidities.

### 2.1. Type 2 Diabetes Mellitus

It has been reported that approximately 285 million adults (aged 20–79 years) suffered from diabetes in 2010. This number is estimated to potentially double by 2030, making this disease the seventh most prevalent cause of death in the world [20]. Although T2DM, which represents nearly 90% of diabetes cases, is primarily observed in the elderly population, an increasing incidence among young people has been observed in recent years, and this increase caused more than 1.6 million deaths worldwide [19]. It should be noticed that T2DM has been diagnosed in nearly 9% of the total adult population in United Kingdom and USA, and more than 25% of the world’s population show a pre-diabetic condition [8]. These pre-diabetic subjects suffer from hyperglycemia associated with high oxidative stress due to the production of reactive oxygen species (ROS) and impaired glucose metabolism [21]. The overabundance of circulating fatty acids, mainly deriving from triglyceride stores in adipose tissue, is one of the key events in the development of insulin resistance [1].

### 2.2. Hypertension and Cardiovascular Diseases

CVDs represent the main cause of death worldwide, with 422.7 million cases (according to a 2015 report), and approximately 18 million deaths [22]. Moreover, it has been estimated that hypertension is diagnosed in 40% of adults (aged >25 years), and the annual expenses due to the patients’ management and the loss of productivity due to heart disease in the USA are estimated to be around 200 billion dollars at least [4]. Noteworthy, Japanese and Korean people have one of the longest average life expectancies and display a lower risk of developing hypertension and cardiovascular diseases than other populations [23,24]. Some epidemiological studies suggested the existence of a causal correlation between these features and the regular consumption of seaweeds [10,25,26].

### 2.3. Obesity, Dyslipidemia, and Nonalcoholic Fatty Liver Disease (NAFLD)

Obesity and dyslipidemia represent two risk factors for the development of MS and coronary disease. Lipid disorders could exacerbate atherosclerosis, leading to chronic heart failure. It has been reported that the 38.2% of the adult USA population suffered from obesity in 2015, whereas in Japan and Korea this percentage was below 6%. Among obese individuals, an increased prevalence (nearly 30%) of Nonalcoholic Fatty Liver Disease (NAFLD) has also been observed with respect to the 9% observed in the general population [8]. NAFLD is a chronic liver disease and is considered the hepatic manifestation of MS [27]. It is characterized by a significant increase in the hepatic lipid content; inflammation; and, in advanced states, liver fibrosis. It is noteworthy that fibrosis may further progress to cirrhosis and hepatocellular carcinoma through the evolution to nonalcoholic steatohepatitis (NASH), a complex disease characterized by hepatocyte injury and/or death, inflammation, and the fibrosis of the hepatic parenchyma [28]. Obese patients with NAFLD commonly show an increase in the plasmatic markers of liver function, such as AST, ALT, and γ-glutamyltransferse (γ-GT) [29]. The alteration of these parameters has also been associated with arterial hypertension, central obesity, hepatic insulin sensitivity, and increased diabetes risk [30].

## 3. Brown Algal Species

*Ochrophyta* (brown seaweeds) comprises 1500–2000 species classified in more than 250 genera [31]. Amongst the three classes of seaweeds (green, red, and brown), brown seaweeds are the most consumed as food (66.5% vs. 33% and 5% of the red and green ones, respectively), and Japan, China, and South Korea represent the three greatest consumers [32]. Brown seaweeds represent an excellent source of nutrients, since they contain high amounts of diverse compounds claimed to exert multiple benefits on health. Therefore, they can be used as bioactive agents in functional foods and nutraceuticals [33,34,35]. At variance with East Asian countries, seaweeds are only rarely present in the European diets, and are still considered as a novel and “exotic” food.

The most studied brown algal species belong to two main orders—i.e., Fucales and Laminariales [31]. Among the Fucales, the principal species are *Ascophyllum nodosum, Fucus serratus, Fucus vesiculosus, Hizikia fusiforme, Sargassum fusiforme, Sargassum horneri, Sargassum mcclurei, Sargassum pallidum*, and *Turbinaria conoides*. The Laminariales order comprises 31 species—for example, *Alaria angusta, Costaria costata, Laminaria japonica, Laminaria cichorioides, Laminaria latissima*, and *Undaria pinnatifida*, amongst others. This order represents the most used and widely exploited worldwide for alginate extraction [36]. Other brown seaweed orders of minor relevance are those belonging to the *Ectocarpales* order, including, for example, *Adenocystis utricularis, Cladosiphon okamuranus, Leathesia difformis, Nemacystus decipiens, Punctaria plantaginea,* and those of the *Dictyotales* order, including *Padina gymnospora* and *Spatoglossum schroederi* [31].

A list of the most relevant *Fucales* and *Laminariales* species, with their most used common names, is reported in Table 1. Among this wide group of brown seaweeds, only a limited number of brown algal species is considered safe for human consumption in Europe—i.e., *Fucus vesiculosus, Fucus serratus, Fucus spiralis, Himanthalia elongata, Undaria pinnatifida, Ascophyllum nodosum, Laminaria digitata, Laminaria saccharina, Laminaria japonica*, and *Alaria esculenta*.

## 4. Bioactive Compounds of Brown Seaweeds

Brown seaweeds are characterized by the presence of different compounds, such as proteins, lipids, carbohydrates, vitamins, and minerals, making them an optimal source of novel ingredients for functional foods. The main bioactive compounds present in brown seaweeds are reported in Table 2. In recent years, polyphenols, sulfated polysaccharides, carotenoids, and polyunsaturated fatty acids (PUFAs) have been evaluated as adjuvants for the treatment and prevention of MS-related diseases [18]. In addition, many useful minerals and indigestible polysaccharides are present in brown seaweeds, the latter being able to affect the digestion and absorption of starch and other complex carbohydrates [37]. Seaweed composition may greatly vary according to multiple factors, such as the algal species, degree of maturity, and growing environment. The seasonal conditions and geographical growth area, as well as other environmental factors such as light, nutrients, salinity, pH, temperature, contaminants, CO_2_ availability, oceanic currents, waves, and biotic interactions, could have a great impact on the composition of bioactive marine compounds, as well as the storage and processing conditions after harvesting [33,35,38,39,40,41]. For example, in Fucus spp. the content of phlorotannins increases according to the water salinity and solar exposure, e.g., during summertime [35].

### 4.1. Minerals and Vitamins

By virtue of their structural and physiological features, brown seaweeds accumulate minerals and vitamins. It has been reported that the content per unit of dry mass of these micronutrients in seaweeds is 10 to 100 times higher than that of terrestrial plants or animal-derived foods [84], and includes both water- and fat-soluble vitamins (C, B1, B2, B9, B12, A, D, E, K) and essential minerals (calcium, iron, iodine, magnesium, potassium, zinc, phosphorus, and selenium) [8]. The presence of these components is extremely variable among algal species. For example, in different brown seaweeds from northern Europe, the vitamin E content is very variable among species, ranging from 1.6 to 122 mg/Kg of dry mass [85].

Peculiar aspects of seaweeds are its high iodine content and low Na/K ratio, although the Na and K contents in seaweeds are generally higher than those reported in terrestrial vegetables [86]. The four brown seaweeds *L. digitata, A. nodosum, H. elongata*, and *U. pinnatifida*, have been reported to display an iodine content of 70, 18.2, 10.7, and 39 mg/Kg wet weight, respectively [87]. Many studies reported a Na/K ratio range of 0.3 to 1.5 in brown seaweeds, which further decreases to 0.3–0.4 in certain Spanish *Laminaria spp*. Just for comparison, other food products—e.g., olives, cheddar cheese, and sausages—have Na/K ratios of 43.6, 8.7, and 4.9, respectively [84,88,89]. The World Health Organization (WHO) recommends the consumption of foods with Na/K ratios as close as possible to 1 or even lower to get a beneficial effect on blood pressure and cardiovascular health [36].

### 4.2. Polyphenols

Brown seaweeds contain high levels of polyphenols, algal secondary metabolites with distinctive antidiabetic, antihyperlipidemic, or anti-inflammatory activities. This wide class of compounds, characterized by the presence of multiple phenol units in their chemical structure, has been extensively studied for their possible clinical use in the management of many diseases, such as, for example, CVDs, diabetes, and hypertension [19]. In particular, brown seaweeds are a source of phlorotannins and bromophenols [90].

#### 4.2.1. Phlorotannins

Phlorotannins are polyphenols exerting multiple ecological roles [35]. The majority of them accumulate in algal physodes and might represent up to 25% of the algal dry weight [91]. They derive from the polymerization of several phloroglucinol units (1,3,5-trihydroxybenzene); are characterized by a high number of hydroxy-groups; are responsible for their high solubility in water; and can be divided into four categories, depending on the linkage between the phloroglucinol units—i.e., fuhalols and phlorethols, fucols, fucophlorethols, and eckols and carmalols [36].

Mechanism of action: Phlorotannins display a wide range of bioactive effects, mainly conducible to the inhibition of the two enzymes, α-glucosidase and α-amylase, which are involved in the intestinal digestion of complex carbohydrates. The digestion of complex polysaccharidic polymers is a necessary step for carbohydrate absorption, since all carbohydrates are absorbed in the form of monosaccharides [92]. Therefore, the inhibition of polysaccharide digestion leads to a drop in their absorption and, consequently, an increase in fecal excretion. Phlorotannin-induced α-glucosidase inhibition could be either noncompetitive (phlorofurofucoeckol-A, 7-phloroeckol, and dioxinodehydroeckol) or competitive (dieckol and eckol) [56,93]. Several studies have demonstrated that many phlorotannins are also able to increase the glucose uptake by skeletal muscles and inhibit the protein tyrosine phosphatase 1B (PTP1B), which regulates the leptin and insulin signaling pathways, thereby improving insulin sensitivity [19,94]. Accordingly, many studies have reported that *A. nodosum* and *F. vesiculosus* display antidiabetic properties due to their phenolic-rich composition, and are able to decrease the postprandial blood glucose levels and insulin peak in various animal models of MS-related disorders [4,95,96]. Moreover, since postprandial “hyperglycemic spikes” could induce micro- and macro-vascular diseases, it is reasonable to hypothesize that these algal phlorotannins could be beneficial for CVD patients [97]. Furthermore, a protective effect against monocyte-associated vascular inflammation and dysfunction has been reported [46]. Moreover, Kwon and collaborators demonstrated that the phlorotannins contained in *E. bicyclis* are able to reduce adipogenesis and lipogenesis and suppress adipocyte differentiation, thus suggesting their efficacy as anti-obesity agents [98].

#### 4.2.2. Bromophenols

Bromophenols are brominated phenolic derivatives with possible anti-diabetic properties by virtue of their inhibitory effect on PTP1B and α-glucosidase [57]. They are characterized by a chemical structure comprising one or more benzene rings, bromine and hydroxyl-substituents, formed by monomers or dimers of brominated 3,4-dihydroxybenzyl alcohol and alkyl ether units [19]. Although they were first isolated from red seaweeds of the *Rhodomelaceae* family, still representing the main source of bromophenols, three derivatives were also isolated from the brown seaweed *Leathesia nana*.

Mechanism of action: Bromophenols are compounds with multiple activities, including an anti-diabetic effect. As recently reviewed, a number of molecular targets have been identified for their involvement in this peculiar bromophenol activity [99]. For example, their inhibitory activity on PTP1B was assessed by Liu and collaborators, further confirming the potential therapeutic efficacy of brown seaweeds for T2DM and obesity treatment [57]. Furthermore, it has been demonstrated that some bromophenols inhibit the enzyme aldose reductase (AR), which converts glucose to sorbitol in the polyol pathway and is known for playing a pivotal role in the prevention of diabetic complications [100].

### 4.3. Polysaccharides

Polysaccharides, deriving from the polymerization of simple sugars by glycosidic bonds, represent the most economically important product obtained from seaweeds. Being mainly localized in the algal cell wall, they confer the flexibility useful for adapting seaweeds to water movements. Their amount ranges from 4% to 76% of the total algal dry mass [92], depending on the species and other factors—e.g., the environmental conditions and season. For example, a polysaccharide content of 37.5%, 65.7%, 69.6%, 35.2%, and 67.8% of dry mass has been reported for *L. japonica, F. vesiculosus, A. nodosum, U. pinnatifida*, and *S. vulgare*, respectively [36].

Cellulose and hemicellulose, which constitute the algal cell wall, represent about 2–10% and 9% of total polysaccharides, respectively. Alginates, fucoidans, and laminarins represent the main polysaccharides of brown seaweeds [101]. Most of these polysaccharides are non-starchy dietary fibers that could help the normalization of blood glucose and cholesterol levels [11,102]. Moreover, the consumption of fiber-rich seaweeds exerts positive effects on appetite and contributes to the 30 g/day Reference Nutrient Intakes (RNI) recommended for fibers [8]. *Laminaria digitata* displays a higher amount of dietary fiber (about 36.12% dry weight) with respect to other brown seaweeds—e.g., *U. pinnatifida* and *F. vesiculosus* [103].

#### 4.3.1. Alginates

Alginates are unbranched indigestible polysaccharides present in brown algal cell walls and intercellular space. They are formed of residues of (1–4)-α-L-guluronic acid and (1–4)-β-D-mannuronic acid, generally as sodium and calcium salts [104]. They represent the main polysaccharides present in brown seaweeds, with a content of 16.9%, 20%, 24%, 32%, 40%, 41%, and 59% dry weight in *S. vulgare, S. longicruris, A. nodosum, S. carpophyllum, L. hyperborean, S. siliquosum*, and *F. vesiculosus*, respectively [36].

Mechanism of action: As with other dietary fibers, the consumption of alginates could delay gastric clearance and increase digestive fluid viscosity and the feeling of satiety [105]. Furthermore, many studies have demonstrated that algal alginates inhibit the digestive enzymes pepsin and pancreatic lipase and decrease the intestinal absorption of glucose, cholesterol, and triacylglycerols [37,104,106]. It has been demonstrated that alginates could decrease pepsin activity by 51–89% on the basis of their polysaccharidic structure [59,104], and it has been hypothesized that the alginate-induced lipase inhibition could be due not only to their direct interaction with the enzyme but also to the formation of a gel able to avoid the enzyme–substrate interaction [60]. Other in vitro studies have demonstrated that alginates inhibit pancreatic lipase in a structure-dependent manner, since the ones with a higher content of guluronic acid displayed the most potent inhibitory effect [104,107]. Taken together, these results support the use of alginates as anti-obesity and anti-MS agents.

#### 4.3.2. Fucose-Containing Sulfated Polysaccharides

Fucoidans are polysaccharides typically found in the cell walls of brown seaweeds, which exert a structural role and prevent the dehydration of algal cells. Their content is highly variable among species, ranging from 6–8%, 3.2–16%, and 3.4–25.7% of dry weight in *Laminaria japonica, Undaria pinnatifida*, and *Fucus vesiculosus* [36]. Frequently, the terms “fucan” and “fucoidan” are interchangeably used, and this is sometimes confusing. For the sake of clarity, fucoidan is a general name that is properly referred to polysaccharides with a variable backbone structure based on neutral sugars and/or uronic acid residues, whereas the term “sulfated fucan” indicates a polysaccharide with sulfated L-fucose residues [31,108].

In recent years, these sulfated molecules have been extensively investigated for their biological activities, including their antioxidant, immunoregulatory, anti-inflammatory, antihypertensive, hypoglycemic, and lipid-lowering effects [63]. These activities are strictly dependent on the chemical composition of these sulfated polysaccharides, which is complex, strongly variable among species, and subject to environmental and seasonal variations [109].

Mechanism of action: It has been reported that fucoidans inhibit lipogenesis and adipocyte differentiation and increase lipolysis, thereby representing an interesting option for obesity treatment. Moreover, fucoidans are well known for their antihypertensive and cardiovascular effects, whose mechanisms have been elucidated and are due to the inhibition of the angiotensin converting enzyme (ACE) and the activation of eNOS-dependent pathways [19]. Moreover, fucoidans are useful in the management of metabolic syndrome and associated diseases, since they reduce hyperglycemia through the inhibition of α-glucosidase and α-amylase, thereby reducing glucose intestinal absorption, and improve the insulin-induced glucose uptake. This effect is due to the capability of fucoidans to modulate relevant pharmacological targets, such as, for example, the AMP-activated protein kinase (AMPK), which is a well-recognized actor and target in metabolic syndrome [110], and the glucose transporter GLUT4 [63,111,112].

#### 4.3.3. Laminarins

Laminarins, polysaccharide-storing components of brown seaweeds, were described for the first time by Schmiedeberg in 1885 in algal cell vacuoles [66]. They are formed by multiple (1,3)-β-D-glucan units, consisting of (1,3)-β-D-glucopyranose residues with 6-O-branching in the main chain and some β-(1,6)-links between chains [67]. Depending on the number of branching of the chains, they could be water-soluble or insoluble.

Mechanism of action: Many useful biological activities could be ascribable to laminarin, such as, for example, anti-cancer, anti-inflammatory, hepatoprotective, and antioxidant properties [68,69]. It has been demonstrated that many laminarin effects are due to the modulation of innate immunity by targeting macrophages. Notably, macrophages are present in metabolic tissues, and are involved in the inflammatory processes occurring in MS [113]. In particular, Lee and collaborators observed that laminarins could exert an in vitro immunostimulatory activity by increasing the expression of gene involved in inflammation and immune response and stimulating the release of inflammatory mediators [70]. Furthermore, laminarins can also act on typical mechanisms involved in MS, since they reduce the systolic blood pressure, cholesterol absorption in the gut, and consequently the cholesterol and total lipid levels both in serum and liver [114].

### 4.4. Terpenoids

#### 4.4.1. Carotenoids

At variance with red and green algae, brown seaweeds are characterized by the presence of the xanthophyll fucoxanthin, which is responsible for their color. Its amount varies among species from 171 mg/kg dry weight (*Fucus spiralis*) to 660 mg/kg dry weight (*Ascophyllum nodosum*) [115].

Mechanism of action: Antidiabetic, anti-obesity, and antioxidant activities have been correlated with fucoxanthin consumption [36,115]. Indeed, fucoxanthin supplementation improved glucose and lipid metabolism and lowered plasma triglycerides and total cholesterol levels in a mouse model of diabetes, reducing insulin resistance [75]. It has been demonstrated that the fucoxanthin extracted from *U. pinnatifida* and its metabolite fucoxanthinol inhibited adipocyte differentiation and hepatic lipid accumulation by downregulating adipogenic and lipogenic factors and upregulating the thermogenic mitochondrial protein UCP-1 in white adipose tissue, thereby exerting an anti-obesity effect [74,115]. Moreover, it has been demonstrated that fucoxanthin displayed hepatoprotective effects. In particular, anti-fibrogenic activity due to the inhibition of hepatic stellate cell activation has been demonstrated in vitro [115,116], and accordingly the inhibition of hepatic oxidative stress, inflammation, and fibrosis has been observed in vivo after the administration of fucoxanthin in a mouse model of NASH [117].

#### 4.4.2. Sterols

Phytosterols are important components of healthy diets, in particular when a hypercholesterolemic effect needs to be pursued. These bioactive algal components can be present either in free form, or esterified with fatty acids glycosylated conjugates [118]. Although cholesterol is widely present in seaweeds of different species, phytosterols—such as, for example, fucosterol—are present in appreciable amounts also in brown seaweeds [118], and have been investigated for their pharmacological effects.

Mechanism of action: Several studies have investigated both in vitro and in vivo the potential health benefits and the underlying pharmacological mechanisms of phytosterols, beyond the well-known cholesterol-lowering effect, obtained by competition with cholesterol upon intestinal absorption [119]. The phytosterol fucosterol exerts hepatoprotective, anti-obesity, anti-diabetic, and antihypertensive activities [46,78,79]. Moreover, it has been demonstrated that it inhibits adipogenesis and adipocytes differentiation by diverse molecular mechanisms [78,79,80]. In particular, the downregulation of Peroxisome proliferator-activated receptor γ (PPARγ) and CCAAT/enhancer binding protein α (C/EBPα) leads to a reduction in lipid accumulation inside adipocytes [79]. The reduction in the absorption of dietary fats by the partial inhibition of the lipolytic enzyme pancreatic lipase is another potential strategy against obesity [120]. As far as the anti-diabetic effect is concerned, this is due to the inhibition of PTP1B, which negatively regulates insulin signaling; AR, which prevents diabetes complications; and α-glucosidase activities. Thumberol, another sterol contained in brown algae, is also able to inhibit significantly PTP1B, thus representing another interesting bioactive component for T2DM and obesity treatment [81].

### 4.5. Alkaloids

Alkaloids are relatively rare in algae. Generally, they present a phenylethylamine and indole or halogenated indole structure. Their biological activities are poorly investigated, even because of their intrinsic toxicity [121].

Mechanism of action: The main concern about the use of alkaloids for MS-related disorders regards their toxicological effects. However, a recent study demonstrated the anti-obesity effects of two indole derivatives isolated from the brown alga *Sargassum thunbergii*, which were able to downregulate the expression of two adipogenic factors—i.e., the nuclear receptor PPARγ and the sterol regulatory element-binding protein 1c (SREBP-1c). These derivatives could also activate the AMPK signaling pathway, thereby decreasing lipid accumulation inside adipocytes and reducing adipogenesis [83].

### 4.6. Lipids and Fatty Acids

Seaweeds contain a low amount of total lipids, generally ranging of from 0.60% to 4.14% of dry weight [101,122]. These lipids are mainly n-3 polyunsaturated fatty acids (PUFAs), such as eicosapentaenoic acid (EPA) and docosahexaenoic acid (DHA), but also some n-6 monounsaturated fatty acids deriving from linoleic and arachidonic acids are present [8]. Although both n-6 and n-3 PUFAs are essential, a dietary consumption unbalanced toward n-6 with respect to n-3 could promotes the development of chronic inflammatory metabolic diseases, such as obesity, NAFLD, and CVDs [123]. In the last two decades, Western countries have observed a progressive increase in foods with a high n-6/n-3 ratio, which has reached 20:1 [124,125], whereas the recommended n-6/n-3 ratio is generally within the range of 2.5:1–4:1 in order to prevent chronic diseases associated with n-6 excessive consumption [126]. Since this ratio inside seaweeds is widely within these recommended values, they could be considered as an optimal dietary lipid source [85].

Mechanism of action: There is conclusive evidence that dietary omega-3 PUFAs can prevent and treat inflammation, which is a hallmark of numerous metabolic disorders. Although many studies have employed the use of fish oil or fish-derived PUFAs, there are a growing number of trials examining the anti-inflammatory effect of algal oils and algal-derived PUFAs. However, conclusive studies dedicated to the effect of the PUFAs obtained from brown seaweeds on MS and related diseases are still lacking. EPA and DHA have been reported to maintain intracellular calcium homeostasis in cardiac cells, and consequently exert an antiarrhythmic effect and reduce the risk of heart disease [71,127], besides upregulating glucose transporters and inhibiting digestive enzymes.

### 4.7. Proteins

The total protein content in seaweeds generally ranges from 5% to 47% of dry mass. Essential amino acids (AAs) represent approximately 42–48% of total AAs. Brown seaweeds display the lowest protein content with respect to red and green ones, and their high content of polyphenols could limit the digestion of these proteins [8]. Nevertheless, an aminoacidic score (an index used to evaluate protein quality) of 1.0, the same reported for egg and soy, for *Undaria pinnatifida*, and 0.82 for *Laminaria* has been reported, to support the fact that seaweeds could represent a viable alternative to animal-derived protein.

Mechanism of action: Despite the low content and digestibility of the proteins contained in brown algae, a number of free AAs and peptides are characterized by key biological activities, such as, for example, ACE inhibitory activity and, consequently, hypotensive effect [82], Akt upregulation, and phosphorylation, processes regulating cardiac glucose metabolism and growth.

### 4.8. Prebiotics

Seaweeds could also represent a source of prebiotics—i.e., “substrates that are selectively utilized by host microorganisms to confer a health benefit” [128]. Probiotics display a number of beneficial effects—e.g., vitamin synthesis, pathogen inhibition, and immune system activation [129]. The bioactive compounds described above—i.e., polyphenols, carotenoids, and PUFAs—could influence the gut microbiota by modulating the microbial activity and composition to elicit biological effects. For example, in a mouse model of obesity, the treatment with an extract of *Saccorhiza polyschides* (12% of indigestible polysaccharides) reduced fat mass and body weight gain due to the alterations of the gut microbiota induced by the fermentation of alginates and fucoidans [129].

## 5. Brown Seaweeds as Functional Food Ingredients for the Management of MS Comorbidities

As described in detail above, brown seaweeds are a high-quality and healthy food by virtue of their low content of lipids and high content of polysaccharides, phytosterols, PUFAs, vitamins, minerals, and other bioactive metabolites. Thus, their use as ingredients of functional food has gained increasing interest in recent years. The global market of seaweeds, which are mainly used in the food, phycocolloid, and fertilizer industries, accounts for 5.5–6 billion US dollars, and it is expected to reach 22.1 billion by 2024 [36,130]. According to the Seafood Source report, the number of new food products containing algal ingredients which have been launched in the European market increased by 147% in 4 years (from 2011 to 2015), confirming the great and increasing interest of Western countries in these ingredients [36]. Even global companies started to develop more sustainable foods using seaweed-based products, as attested by the project proposed by a famous global furniture company for the addition of vegetables and, in particular, algal components to beef meatballs and pork hotdogs [8].

Many studies on products with added brown seaweeds demonstrated that their supplementation significantly improved the nutritional value by increasing the content of dietary fibers, n-3 PUFAs, and minerals (e.g., Ca, Mg, and K), reducing the Na/K ratio and ameliorating the lipidic profile [8]. Since some MS-related manifestations—i.e., heart disease and obesity—are sustained by a high consumption of Na, saturated fats, and artificial additives, adding brown seaweeds to meat- and grain-based products has been proposed as a useful strategy to prevent their development. A selection of studies investigating the use of brown algae as functional food ingredients and demonstrating their beneficial effect on MS comorbidities is reported in Table 3. Accordingly, a variety of foods have been reformulated with the addition of brown seaweeds in order to reduce and/or remove the original low-quality ingredients and replace them with healthier bioactive components. Indeed, the peculiar composition of algae, as explained and detailed above, provides natural antioxidant and preservative properties to these seaweed-supplemented foods [131].

The most promising attempts to develop brown algal-fortified functional foods useful for MS management have been obtained by adding *U. pinnatifida, L. japonica*, and *H. elongata* to meat-based and grain-based products. For example, Astorga-España and collaborators studied the incorporation of Chilean brown seaweeds to dishes commonly prepared in that region—e.g., hamburgers, bread, breadsticks, and fritters. This led to dishes with a greater fiber and n-3 PUFA content than conventional foods, thereby characterized by a better nutritional profile [144]. The incorporation of *U. pinnatifida* and *H. elongata* into several meat-based products (pork frankfurters, beef burgers, and restructured poultry steaks) reduced by 50% to 75% the NaCl content with respect to conventional products and improved the lipoprotein metabolism in animal models [133,134,137,138,145,146,147,148]. In a rat model of hypercholesterolemia, the introduction of *H. elongata* to pork meat was able to reduce the plasma cholesterol levels by upregulating glutathione reductase (*GR)*; superoxide dismutase (*SOD)*; and *CYP7A1*, which is involved in cholesterol metabolism, and downregulating catalase (*CAT*) and glutathione peroxidase (*GPx*), finally exerting anti-oxidant and hypocholesterolemic effects [133]. Accordingly, it has also been demonstrated that wakame-enriched restructured meats positively affected glutathione levels, increased the GR and SOD activity, thereby exerting an antioxidant effect [134,135].

The brown seaweed *Himanthalia elongata* was added to beef patties, increasing the dietary fiber content, reducing the fat content, and improving the antioxidant activity [149]. The same effect was obtained by the addition of fucoxanthin extracted from *Cystoseira barbata* to turkey meat sausages, which also led to the inhibition of lipid peroxidation and ACE activity [139].

Some studies have reported the beneficial effect of the brown seaweed addition to grain-derived food products. In particular, the addition of *U. pinnatifida* (5–30% w/w) to uncooked pasta was reported to enhance the antioxidant properties by virtue of the higher phenolic content [142]. *H. elongata*-enriched breadsticks have a greater content of total dietary fibers and phenols with respect to their conventional counterparts, thus improving antioxidant capacity [140]. Moreover, a single blind crossover trial conducted by Hall and collaborators demonstrated that the addition of *A. nodosum* to bread products decreased the energy intake after a meal, whereas no differences were registered in the blood glucose and cholesterol plasma levels [141]. These authors pointed out the need of a long-term study to investigate the potential use of *A. nodosum*-enriched bread in overweight subjects and deepen its mechanism of action. Another study investigated the effect of a combination of *U. pinnatifida* and *Ceratonia siliqua* L. in functional snacks [143]. In a rat model, the anti-inflammatory and anti-hypertensive effects of these algal-enriched snacks were observed, suggesting its potential use for MS management.

Besides meat- and grain-based foods, many other studies have investigated the effect of seaweed addition on the nutritional value and antioxidant properties of dairy products, fish, desserts, mayonnaise, sauces, and fermented products. The current trends in enhancing the antioxidant activity of by incorporating natural bioactive compounds are presented. Additional in vivo preclinical and clinical studies are needed to validate their application for MS management [150,151,152].

## 6. Bioavailability of Brown Seaweeds

Little is known about the bioavailability of the bioactive compounds present in both brown seaweeds and their derived products, mainly because of the heterogenicity of the algal matrix. In vitro digestion studies would help to assess the stability of seaweed components both in extracts and within the seaweed matrix [153], but they should also be implemented by in vivo/ex vivo evaluations and human studies, since the absorption and metabolism could be strongly affected by the species in which the evaluations have been carried out [154].

Since biological activity is strictly related to bioavailability and metabolism, the evaluation of the “absorption, distribution, metabolism, and excretion” (ADME) profile of brown algal bioactive compounds in humans, together with the determination of possible interactions with food or drugs [155], is of primary importance. A study carried out by Corona and collaborators investigated the digestion and absorption of phlorotannins extracted from *A. nodosum* after oral administration to healthy volunteers and their effect on some inflammatory markers [156]. These authors identified some phlorotannin oligomers produced after digestion and colonic fermentation and detected multiple unconjugated and conjugated metabolites (glucuronides and/or sulphates) in both urine and plasma (after 6–24 h), concluding that their absorption and metabolism occurred in the large intestine. Hydroxytrifuhalol A, 7-hydroxyeckol, and C-O-C dimer of phloroglucinol, which were present in the administered extract, were also detected in urine. They also correlate the presence of phlorotannin metabolites in the blood with the reduction in circulating IL-8, thus suggesting that this cytokine can be considered a putative target of efficacy.

Another study by Baldrick and collaborators assessed the bioavailability and activity of the polyphenols extracted from *A. nodosum*, analyzing their 24 h urinary excretion in 78 overweight and obese individuals, finding in urine different conjugates of polyphenolic compounds [157].

As stated before, algal polysaccharides, (alginates, fucoidans, and laminarins) are considered dietary fibers, characterized by β(1→4) bonds that human digestive enzymes cannot disrupt. However, resident colonic bacteria partially ferment them into short chain fatty acids (SCFAs). In animal models, it has been observed that fucoidan could accumulate in the jejunal and hepatic cells, thus demonstrating that it could be partially absorbed [158]. This observation was also confirmed by clinical studies that detected unchanged fucoidan in human serum and urine of algal eaters, although the mechanism of absorption needs to be fully explored [159,160].

Like dietary lipids and lipid-soluble vitamins, the algal carotenoid fucoxanthin is probably absorbed in the proximal half of the small intestine [115], and its absorption could be increased by the presence of other oils and lipids [76]. Although not demonstrated, fucoxanthin can enter via facilitated diffusion into the enterocytes using the scavenger receptor class B type 1 (SR-B1) as a carrier, like other carotenoids [161]. In mammals, fucoxanthin is metabolized to fucoxanthinol and then in amarouciaxanthin A, as demonstrated by the accumulation of these metabolites in mouse plasma and tissues after the oral administration of fucoxanthin extracted from *L. japonica* and *U. pinnatifida* [162,163]. As reviewed by Viera and collaborators, only a few clinical studies demonstrated the bioavailability of fucoxanthin, although this was low and strongly affected by the presence of dietary fibers in the food matrix, together with fast first-pass metabolism, and a low affinity to intestinal transporters [164].

An interesting meta-analysis by Xi and collaborators analyzed 364 research papers in order to identify the putative biomarkers of food intake (BFIs). They proposed seven candidate BFIs for evaluating brown seaweed absorption—i.e., hydroxytrifuhalol A, 7-hydroxyeckol, C-O-C dimer of phloroglucinol, diphloroethol, fucophloroethol, dioxino-dehydroeckol, and fucoxanthinol—as well as their glucuronides or sulfate esters [165]. It can be suggested that these compounds may be useful for correlating the observed biological effects with the absorption of bioactive compounds from seaweed extracts and seaweed-containing products. Nevertheless, the use of these compounds as markers of efficacy should be validated.

## 7. Clinical Studies Investigating Brown Seaweeds for MS Treatment

Many studies investigating the activity of brown seaweeds are performed by in vitro or in vivo evaluations on cell lines or animal models, respectively, providing valuable information regarding the identification of the bioactive components responsible for the effect and the signaling pathways involved. For example, a number of in vivo animal studies have demonstrated the anti-diabetic and hypoglycemic efficacy of brown seaweeds [4,95,96,166,167]. The principal mechanism involved in this activity is the inhibition of digestive enzymes (e.g., α-amylase, α-glucosidase, lipase, PTP1B), which causes a reduction in dietary fat and glucose absorption, and the hepatic gluconeogenesis. These effects are mainly due to algal phlorotannins, fucoxanthin, polyphenols, and polysaccharides [168,169]. Nevertheless, clinical studies are a fundamental step for the evaluation of the effective and safe use of algal extracts and are necessary to identify the molecular identity of effective compounds. *E. cava, U. pinnatifida, A. nodosum*, and *F. vesiculosus* are the most investigated brown seaweeds for the management of MS-related disease. Table 4 describes the principal clinical studies dealing with the efficacy of brown algae in MS comorbidities.

### 7.1. Ascophyllum Nodosum and Fucus Vesiculosus

*A. nodosum* and *F. vesiculosus* are two of the most exploited brown seaweeds for the treatment of hyperglycemia and diabetes, since it is well-known that they inhibit the two digestive enzymes α-amylase and α-glucosidase in a dose-dependent manner and decrease postprandial blood glucose levels of animal models of hyperglycemia and NASH [95,96]. The first clinical trial investigating the antidiabetic properties of these two seaweeds was conducted by Paradis and collaborators in 2011 [112]. They examined the effect of a commercial extract of *A. nodosum* and *F. vesiculosus* administered to 23 subjects 30 min before a carbohydrate meal. A single administration of the algal extract had no effect on the glucose response but caused a decrease in the insulin iAUC and an increase in the Cederholm index, suggesting an improvement in insulin sensitivity. Another study enrolling overweight and obese patients demonstrated that a 6-month supplementation with the same commercial extract of *F. vesiculosus* and *A. nodosum* with the addition of chromium picolinate significantly reduces the waist circumference, plasma levels of glucose, and insulin and HOMA index, suggesting an improvement in the insulin sensitivity status [172]. In addition, a reduction in HbA1c, fasting plasma glucose and insulin, postprandial plasma glucose, and HOMA-IR was observed after the administration of the same nutraceutical combination for 6 months to 65 dysglycemic patients [171]. Moreover, nearly 20% of the treated patients retuned to a normal glycemic status, and none of the placebo-treated patients. A reduction in high-sensitivity C-reactive protein (Hs-CRP) and TNF-α levels was also observed in treated patients. A study investigating the effect of an extract of polyphenols contained in *A. nodosum* on DNA damage and oxidative stress and inflammation demonstrated a decrease in lymphocyte DNA damage in obese but not in overweight subjects, suggesting a selective improvement of obesity-related inflammatory status [157]. These studies further confirmed the capability of *A. nodosum* and *F. vesiculosus* to the ameliorate insulin sensitivity, inflammation, and glycemic status of dysglycemic patients.

A randomized, placebo-controlled, double-blind, parallel group trial on subjects commonly experiencing postprandial drowsiness demonstrated that the administration of a brown seaweed extract (equivalent to 10 g of dried seaweed) was able to improve cognitive function after a high-carbohydrate lunch. This effect was due to the reduced glycemic response to the food, consequent to the inhibition of α-amylase and α-glucosidase, as hypothesized also by previous studies that linked the cognitive beneficial effects to low-glycemic index (GI) foods [170]. This study is pioneering, since it represents the first attempt at demonstrating cognitive benefits due to seaweed extract administration.

Murray and collaborators investigated the effect of a single administration of two doses of a polyphenol-rich *F. vesiculosus* extract (500 mg and 2000 mg) to healthy adults, without observing any differences in the iAUC and the postprandial peak of glycemia and plasma insulin with respect to placebo (2 g of cellulose fiber). This study demonstrated that healthy Asian people have a higher postprandial insulin response, without any sign of glucose tolerance, with respect to non-Asian subjects, which could not be improved by a single dose of algal polyphenols [173]. The same research group proposed a protocol for a double-blind randomized controlled trial to be performed on hypercholesterolemic overweight or obese patients in order to evaluate the effect of 12-week supplementation with a *F. vesiculosus* extract rich in polyphenols and fucoidans, but the results have not yet been published [185].

### 7.2. Ecklonia Cava

The brown seaweed *Ecklonia cava* is recognized as a rich source of polyphenols/pholotannins—e.g., dieckol—which could exert glucose and lipid-lowering effects [50,94,168]. Three large clinical double-blind placebo-controlled randomized trials investigated the antidiabetic, anti-obesity, and hypocholesterolemic properties of the polyphenols extracted from *E. cava*. The study of Shin et al. examined the effect of E. cava phlorotannins in 97 Korean overweight male and female adults (BMI 26.5 ± 1.6 kg/m²) [175]. After a 12-week treatment, a reduction in the BMI waist circumference, body fat ratio, waist/hip ratio, total cholesterol, LDL cholesterol, total cholesterol/HDL ratio, and atherogenic index was observed. Moreover, a high dosage of phlorotannins (144 mg/day) was able to significantly decrease the plasma glucose levels and systolic blood pressure with respect to the control without causing any adverse effect. Another study investigated the effect of a 12-week administration of a dieckol-rich extract (500 mg/3 times day) to 80 pre-diabetic individuals [94]. The dieckol-rich *E. cava* extract significantly decreased the postprandial glucose, insulin, and C-peptide levels after 12 weeks, further confirming its promising activity. Choi and collaborators investigated the lipid-lowering effect of an extract of this seaweed in hypercholesterolemic subjects (plasma total cholesterol > 200 mg/dL and LDL cholesterol > 110 mg/dL) [176] and observed a significant decrease in both the total and LDL cholesterol in algal extract-treated subjects with respect to the controls.

It is noteworthy that no significant adverse reactions could be observed in these studies due to 12-week algal administration, but more long-term studies need to be conducted to confirm the safety and extent of effectiveness in humans.

### 7.3. Undaria Pinnatifida

The brown seaweed *U. pinnatifida* is native to Eastern Asia and has been exported to many other countries, including Europe, Australia, and New Zealand. It is also known as Wakame or sea mustard and is among the most commonly consumed seaweeds in Korea. A study of Kim evaluated the effect of a 4-week supplementation with dietary fibers obtained from sea tangle (*L. japonica*) and sea mustard (*U. pinnatifida*) in T2DM patients [102]. These authors observed that the intake of total dietary fibers was 2.5 times higher in seaweed-treated patients, and that this increase was inversely correlated to the fasting and postprandial blood glucose levels. Additionally, triglycerides and LDL cholesterol serum levels were decreased by the seaweed administration. Moreover, an increase in antioxidant enzyme activities (CAT and glutathione peroxidase) was observed in these patients, which correlated to a lower level of thiobarbituric acid-reactive substances (TBARS) in erythrocytes with respect to controls. In summary, the supplementation with *Undaria pinnatifida* and *Sacchariza polyschides* improved glycemic control and lowered blood lipids and antioxidant defenses.

A large double-blind randomized placebo-controlled study preformed on 151 obese, premenopausal, non-diabetic females with and without NAFLD analyzed the effect of a preparation containing a brown seaweed extract rich in fucoxanthin over a 16-week period [180]. This product reduced body weight, body and liver fat content, and serum TG and C-reactive protein. In addition to the reduction in these parameters, a significant decrease in waist circumference and liver enzymes (ALT, AST, γ-GT) and an increase in resting energy expenditure was also observed in the treated NAFLD patients.

Another study investigated the enrichment of a test meal enriched with Wakame and Mekabu, two nutritionally identical preparations obtained from *U. pinnatifida* characterized by different viscosities. The Mekabu meal significantly reduced the postprandial blood glucose levels (30 min) and glucose AUC, modifying the high glycaemic index of white rice, thus confirming the usefulness of this brown seaweed in controlling T2DM [177].

A study conducted in Japan on 36 elderly hypertensive outpatients treated with *U. pinnatifida* demonstrated a decrease in both systolic and diastolic pressure and an 8% reduction in hypercholesterolemia after 4–8 weeks of treatment [178]. A similar result was obtained in a randomized, double-blinded, placebo-controlled trial performed on patients with at least one MS symptom. This study concludes that the consumption of at least 4 g/day of *U. pinnatifida* is associated with a reduction in MS prevalence [179].

### 7.4. Laminaria Species

*Laminaria* species—e.g., *L. japonica*—are widely used brown seaweeds, especially in Asian countries, well known for their high content of indigestible fibers and fucoxanthin. A study of Hitoe and Shimoda examined the effect of a 4-week administration of fucoxanthin extracted from Laminaria in 50 subjects aged 20–59, with a BMI of ≥26–30 kg/m^2^ and waist circumference of ≥90 cm for females and ≥85 cm for males. Fucoxanthin was able to decrease the body weight, BMI, and abdominal visceral fat, thus improving moderate overweight states, without displaying any adverse effect [181].

In addition to fucoxanthin, the effect of the fucoidan extracted from *Laminaria* species was also evaluated in overweight/obese adults [182]. The oral administration of 500 mg of fucoidan for 3 months induced a significant decrease in the diastolic blood pressure and LDL cholesterol and an increase in the plasma insulin and HOMA index, thus improving insulin secretion and resistance.

Another clinical study investigated the antioxidant potential of fermented *Laminaria japonica* in healthy individuals with high levels of γ-GT (<132 U/L). A one-month administration of this seaweed significantly reduced the serum malondialdehyde (MDA) and γ-GT and significantly increased the levels of CAT and SOD, thereby improving the liver antioxidant defense [183]. This effect could be useful for managing oxidative stress-related hepatic damage and cardiovascular disease and further confirmed the antioxidant, anti-diabetic, and anti-hypertensive properties of Korean rice wine fermented with this brown seaweed which had been previously observed in vitro [186].

## 8. Future Trends

Brown seaweeds represent a sustainable and low-cost source of a variety of bioactive compounds, displaying multiple beneficial effects for human health. Being a low-caloric food free of saturated fat, they could represent an excellent alternative for the intake of n-3 FAs derived from fish and are suitable for many functional food applications. For these reasons, there is a growing interest in their use for medicinal applications, mainly for lifestyle-related diseases such as T2DM, hypertension, obesity, and cardiovascular diseases, all of which are associated to MS development. To support this evidence, the global seaweed market is evaluated to rise from $10.4 billion as of 2015 to $14.7 billion as of 2021 [8].

As extensively described above, the mechanisms by which bioactive algal components could exert their effect have been investigated using many cell lines and animal models—e.g., adipocytes, high fat-fed rats or mice, and genetically modified mice [4,19,95,96,187]. Furthermore, a number of controlled clinical trials conducted in men and women adults and older have demonstrated in the last few years the potential of the use of brown seaweeds for the management of MS comorbidities (Table 4), further confirming the results obtained by in vitro and in vivo studies.

MS comorbidities were traditionally considered as diseases of adulthood; nevertheless, the increasing incidence of obesity between children and adolescents has raised the need for safe and effective agents to be used at different ages to prevent obesity. Furthermore, juvenile obesity could predispose one to T2DM, hypertension, dyslipidemia, NASH, left ventricular hypertrophy, obstructive sleep apnea, psychosocial complications, and orthopedic problems in adult life [1,19,188]. Interestingly, a Japanese interventional study investigated the effect of the red seaweed nori on blood pressure in children aged 4 to 5 years, observing a decrease in diastolic blood pressure in boys [189]. The authors thus suggest that nori seaweed might represent a preventive intervention for treating elevated blood pressure in childhood. This study represents the first attempt to use seaweeds in young people as a supplement for MS comorbidities, and could open a new perspective also for the future development of brown seaweed use.

Furthermore, seaweeds have been used for enhancing the antioxidant activity of gluten-free baked products, and this application opens new avenues in the enrichments of foods dedicated to coeliac patients [190].

## 9. Conclusions

In conclusion, brown seaweeds have demonstrated a great potential as food supplements for MS management. However, some issues still need to be deepened to improve the knowledge of their ADME in humans, find validated indexes of algal absorption, and obtain reliable information on their efficacy and long-term safety.

## Figures and Tables

**Table 1:** List of some brown algal species belonging to *Fucales* and *Laminariales* (Algabase database) with the common names used in the literature, when available.

Species	Selected Common Names
**Fucales**	
*Ascophyllum nodosum* (Linnaeus) *Le Jolis*	Yellow Tang, Knotted wrack, Knobbed Wrack
*Cystoseira barbata* (Stackhouse) *C. Agardh*	
*Cystoseira crinita*	
*Durvillaea antarctica* (Chamisso) *Hariot*	Bull kelp, Cochayugo
*Fucus serratus* *Linnaeus*	Serrated wrack, Saw Wrack, Toothed Wrack
*Fucus spiralis Linnaeus*	Jelly bags, Spiral wrack
*Fucus vesiculosus Linnaeus*	Paddy Tang, Sea ware, Bladder, Rockweed, Bladder wrack
*Himanthalia elongata* (Linnaeus) *S. F. Gray*	Sea thong, Sea spaghetti
*Hizikia fusiformis or Sargassum fusiforme* (Harvey) *Setchell*	Hai tso, Hijiki
*Sargassum crassifolium J. Agardh or Sargassum aquifolium* (Turner) *C. Agardh*	Binder’s Sargassum weed
*Sargassum fluitans* (Borgesen)	
*Sargassum horneri* (Turner) *C. Agardh*	Akamoku
*Sargassum thunbergii* (Mertens ex Roth) *Kuntze*	
*Silvetia compressa* (J. Agardh)	
**Laminariales**	
*Alaria angusta*	
*Alaria esculenta*	Bladderlocks
*Costaria costata* (C. Agardh)	Sujime
*Ecklonia arborea* (Areschoug)	
*Ecklonia cava* Kjellman	Kajime
*Ecklonia kurome*	
*Ecklonia stolonifera*	
*Egregia menziesii* (Turner) Areschoug	Feather boa, Boa kelp
*Eisenia bicyclis* (Kjellman) Setchell	Arame, Kajimi, Sagarame
*Laminaria cichorioides or Saccharina cichorioides* (Miyabe)	
*Laminaria digitate* (Hudson) J. V. Lamouroux	Kombu
*Laminaria hyperborea* (Gunnerus) Foslie	Cuvie, Forest Kelp, Kelpie
*Laminaria japonica Areschoug or Saccharina japonica* (Areschoug)	Hai Dai, Sea Tangle, Makombu Tasima, Royal kombu
*Laminaria saccharina or Saccharina latissima* (Linnaeus)	Sea belt, Sweet Wrack, Sugar Wrack, Karafuto Kombu
*Macrocystis pyrifera* (Linnaeus) C. Agardh	Giant Kelp, Sea Ivy
*Undaria pinnatifida* (Harvey) Suringar	Qun dai cai, Wakame, Sea mustard, Miyok

**Table 2:** Principal classes of brown seaweed compounds and metabolites with their relative biological effects in the context of MS comorbidities.

Algal Component	Bioactive Compounds	Main Molecular Pathways	Refs.
**Polyphenols**			
**Phlorotannins**	2,5-dihydroxybenzoic acid, Phloroglucinol, Ishophloroglucin A	Inhibition of α-glucosidase, α-amylase and lipase, 3-hydroxy-3-methylglutaryl-CoA (HMGCoA) reductase.	[37,42,43,44,45,46,47,48,49,50,51,52,53,54,55,56]
	**Fuhalols and Phlorethols:** Octaphlorethol A, Triphlorethol-A	Downregulation of adipogenic specific proteins: PPARγ, SREBPs, C/EBPα, and adiponectin.	
	**Fucols**	Activation of Akt and AMPKα signaling.	
	**Fucophlorethols:** Phlorofucofuroeckol A	Downregulation of perilipin, TNFα, FABP4, FASN, FATP1, Leptin, and acyl-CoA synthetase 1.	
	**Eckols and carmalols**: Dieckol, 6,6′-Bieckol, 8,8′-Bieckol, 2-O-(2,4,6-trihydroxyphenyl)-6,6′-bieckol, Phloroglucinol-6,6-Bieckol, 2,7″-Phloroglucinol-6,6′-bieckol, Pyrogallol- Diphlorethohydroxycarmalol Eckol, Dioxindehydroeckol7-phloroeckol	ACE inhibition.	
		Up-regulation of GLUT4.	
		Downregulation of phosphoenolpyruvate carboxykinase (PEPCK), glucose-6-phosphatase (G6Pase), and gluconeogenesis-related enzymes.	
		Increase glycerol secretion.	
		Increase eNOS phosphorylation.	
**Bromophenols**	3,4-dibromo-5-(methoxymethyl)-1,2-benzenediol,	Inhibition of PTP1B activity.	[57,58]
	2-methyl-3-(2,3-dibromo-4,5-dihydroxy)-propylaldehyde		
	3-(2,3-dibromo-4,5-dihydroxy-phenyl)-4-bromo-5,6-dihydroxy-1,3-dihydroiso-benzofuran		
**Polysaccharides**			
**Alginates**		Inhibition of α-amylase, pancreatic lipase and pepsin.	[42,59,60]
**Sulphated fucans**		Inhibition of α-amylase and ACE, protective effects against ROS.	[61]
**Fucoidans**		Inhibition of α-glucosidase and α-amylase.	[62,63,64,65]
		Inhibition of ACE.	
		Downregulation of hemoglobin A1c (HbA1c) levels.	
		Increase NO production, eNOS activation, and Akt phosphorylation.	
		Activation of PI3K/Akt/eNOS-dependent pathways.	
		Inhibition of adipocyte differentiation and basal lipolysis.	
		Acceleration of the mitochondrial β-oxidation, peroxisomal oxidation or degradation.	
		Modulation of RCT-related protein expression.	
		Upregulation of superoxide dismutase and catalase.	
**Laminarins**		Upregulation of STAT1, STAT3, c-Jun, c-Fos, and COX-2 in macrophages.	[66,67,68,69,70]
**Lipids and Fatty acids**	n-3 fatty acids	Inhibition of α-glucosidase and α-amylase.	[71]
		Up-regulation of GLUT1 and GLUT4.	
		Increase insulin sensitivity.	
**Terpenoids**			
**Carotenoids**	Fucoxanthin	Decrease in lipid accumulation.	[38,72,73,74,75,76]
		Inhibition of advanced glycation end product formation.	
		Inhibition of PTP1B activity.	
		Increase AGE formation.	
		Upregulation of PPARα, p-ACC, and CPT-1; modulation of IRS-1/PI3K/AKT and AMPK signaling.	
		Downregulation of adipogenic and lipogenic factors, such as CCAAT/C/EBPα, PPARγ, fatty acid-binding protein 4, diglyceride acyltransferase 1, and lysophosphatidic acid acyltransferase-θ.	
		UCP-1 upregulation in white adipose tissue.	
**Sterols**	Fucosterol, Thunberol	Inhibition of PTP1B, human recombinant aldose reductase (HRAR), and α-glucosidase activity.	[46,77,78,79,80,81]
		Downregulation of PPARγ and C/EBPα expression.	
		Inhibition of advanced glycation end product formation.	
**Peptides**		Inhibition of α-glucosidase and α-amylase.	[82]
		Akt upregulation and PI3K/AKT phosphorylation.	
		ACE inhibition.	
**Alkaloids**	Indole-2-carboxaldehyde	Downregulation of the SREBP-1c, PPARγ C/EBPα; inhibition of adipogenesis through AMPK activation.	[83]
	Indole-6-carboxaldehyde	Inhibition of adipocyte differentiation and lipid accumulation.	

**Table 3:** Principal studies investigating the potential use of brown seaweeds as functional food ingredients to improve MS.

Functional Food	Functional Ingredient	Observed Effect	Refs.
**Meat-Based Products**			
Low-salt pork emulsion systems	5.6% dry matter *H. elongata or U. pinnatifida*	Increase in n-3 PUFA content.	[132]
		Improvement of n-6/n-3 PUFA ratio and thrombogenic index.	
		Increased concentrations of K, Ca, Mg, and Mn.	
		Increase in antioxidant capacity.	
Restructured pork meat	5% powder *H. elongata* or *U. pinnatifida*	Modification of lipogenic/lipolytic enzyme expression:	[133,134,135]
		Downregulation of acetyl fatty acid synthase (FAS) and hormone-sensitive lipase (HSL) and upregulation of CoA carboxylase (ACC).	
		Decrease in caspase-3 activity.	
		Improvement of the hepatic antioxidant status, increasing total and reduced glutathione and gene expression of CYP7A1, GR, and Cu,Zn-SOD and decreasing the redox index.	
		Decrease cholesterol plasma level in rat models of hypercholesterolemia.	
Pork/chicken patties	*L. japonica* (replacing pork/chicken in equal amount)	Decrease in postprandial glucose blood levels in borderline-hypercholesterolemic patients.	[136]
Frankfurters	5.5% *H. elongata*	Aid in the maintenance of normal blood pressure due to the reduced sodium content.	[137,138]
		Improvement of n-6/n-3 PUFA ratio and the maintenance of normal blood cholesterol levels due to the replacement of saturated fats with unsaturated, with at least 70% of the fatty acids	
		Contribution of EPA and DHA to the maintenance of normal function of the heart.	
Turkey meat sausages	Fucoxanthin from *C. barbata*	Improvement of antioxidant activity.	[139]
		ACE inhibition.	
**Grain-Based Products**			
Bread	8% (w/w) *H. elongata* and *U. pinnatifida*	Improvement of antioxidant activity in DPPH, ORAC, and TEAC assays.	[140]
Bread	4% *A. nodosum*	Decrease in energy intake after meal.	[141]
Pasta	10% *U. pinnatifida*	Improvement of amino acid, fatty acid profile, and nutritional value.	[142]
Functional snacks	1/5 combination of *U. pinnatifida* and *Ceratonia siliqua* L.	In vitro TG-lowering effect and downregulation of DGAT2.	[143]
		Anti-hypertensive effect in rats with MS.	

**Table 4:** Principal clinical studies investigating the effect of brown seaweeds in MS comorbidities.

Brown Seaweeds	Bioactive Compound	Study Design and Population	Observed Effect	Refs.
*A. nodosum and F. vesiculosus*	Polyphenols, fibers, minerals	Double-blind, placebo-controlled, cross-over randomized trial with 23 men and women (18–60 years) with BMI 20–30 Kg/m^2^.	Decrease in insulin iAUC. Increase in the Cederholm index of insulin sensitivity.	[112]
*A. nodosum and F. vesiculosus*	Phlorotannins	60 men and women (18–65 years).	Improvement of postprandial cognitive performance and drowsiness.	[170]
*A. nodosum and F. vesiculosus*	Polyphenol extract (titrated to 20%)	65 dysglycemic patients.	Reduction in HbA1c, fasting plasma glucose, postprandial plasma glucose, fasting plasma insulin, high sensitivity C-reactive protein, and HOMA-IR. Improve insulin sensitivity and glycemic status.	[171]
*A. nodosum and F. vesiculosus*	Polyphenol extract (titrated to 20%)	50 men and women (18–60 years), 44 overweight and 6 obese.	Reduction in waist circumference, plasma glucose, and insulin and HOMA index.	[172]
*A. nodosum*	Polyphenols (phlorotannins)	Double-blind, randomized, placebo-controlled crossover trial with 80 subjects (30–65 years) with BMI ≥ 25 Kg/m^2^.	Decrease in DNA damage in obese subjects. No significant changes in CRP, inflammatory cytokines, and antioxidant status.	[157]
*F. vesiculosus*	Polyphenols	Double-blind, placebo-controlled, randomized, cross-over trial with 38 volunteers (26 non-Asian, 12 Asian, 19–56 years).	No lowering effect on postprandial glucose or insulin responses in healthy subjects. Different insulin sensitivity in Asian subjects.	[173]
*E. cava*	Dieckol	Double-blind, placebo-controlled, randomized trial with 80 men and women (20–65 years) with a fasting glucose between 100 and 180 mg/dL.	Decrease in postprandial glucose, insulin, and C-peptide levels.	[174]
*E. cava*	Polyphenols. Including dieckol, 8,8’-bieckol, 6,6’-bieckol, and phlorofurofucoeckol A	Double-blind, placebo-controlled, randomized trial with 97 men and women (19–55 years) with BMI 24–29 Kg/m^2^.	Decrease in BMI, body fat ratio, waist circumference, waist/hip ratio, total cholesterol, low-density lipoprotein (LDL) cholesterol, total cholesterol/high-density lipoprotein (HDL), cholesterol, and atherogenic index. High dosage showed also significant decreases in serum glucose and systolic blood pressure.	[175]
*E. cava*	Polyphenols (dieckol)	Double-blind, placebo-controlled, randomized trial with 80 healthy subjects (19–80 years) with total cholesterol > 200 mg/dL or of LDL cholesterol > 110 mg/dL.	Decrease in total cholesterol and LDL cholesterol levels.	[176]
*U. pinnatifida and L. japonica*	Indigestible polysaccharides dietary fiber	20 T2D patients (men and women, 40–70 years).	Improvement of blood glucose levels, serum TG decrease. Increase in HDL cholesterol and activity of CAT and glutathione peroxidase.	[102]
*U. pinnatifida*	Fresh Wakame or Mekabu	Randomized, crossover study with 12 healthy adults (men and women).	Reduction in plasma glucose levels, due to the improvement of glycemic index of foods.	[177]
*U. pinnatifida*	Dried Wakame powder	36 elderly outpatients with hypertension.	Decrease in systolic and diastolic blood pressure. Improvement of hypercholesterolemia.	[178]
*U. pinnatifida*	Dried algal powder	27 patients (men and women) with at least one symptom of MS.	Decrease in systolic blood pressure and waist circumference.	[179]
*U. pinnatifida*	Fucoxanthin	Double-blind, randomized, placebo-controlled study of 115 obese, premenopausal, non-diabetic women with and without NAFLD.	Decrease in body weight, waist circumference, body and liver fat content. Improvement in liver function tests and resting energy expenditure.	[180]
*Kelp Laminaria*	Fucoxanthin	Randomized, double-blind, placebo-controlled crossover trial with 50 men and women (20–59 years) with a BMI of > 26–30 Kg/m2 and waist circumference of ≥90 cm (women) and ≥85 cm (men).	Decrease in body weight, BMI, and visceral fat.	[181]
*L. japonica*	Fucoidan	Double-blind, placebo-controlled, randomized trial with 25 overweight or obese adults (30–60 years).	Decrease in diastolic blood pressure and LDL-C. Increase in insulin levels, HOMA β-cell, and HOMA IR.	[182]
Fermented *L. japonica*	5.56%-aminobutyric acid (GABA)	Randomized, controlled trial with healthy subjects with high levels of γ-GT (< 132 U/L).	Decrease in serum γ-GT and malondialdehyde. Reduction in oxidative stress. Increases antioxidant activity of CAT and SOD.	[183]
*S. horneri*	Fucoxanthin	Single-blinded and randomized study with 60 normal-weight and obese Japanese adults with a BMI > 22 Kg/m^2^.	Decrease in HbA1c levels.	[184]

## References

[1] Eckel R.H., Grundy S.M., Zimmet P.Z. (2005). The metabolic syndrome. Lancet.

[2] WHO (2014). Global Status Report on Noncommunicable Diseases. http://www.who.int/nmh/publications/ncd-status-report-2014/en/.

[3] Waltenberger B., Mocan A., Šmejkal K., Heiss E.H., Atanasov A.G. (2016). Natural Products to Counteract the Epidemic of Cardiovascular and Metabolic Disorders. Molecules.

[4] Gabbia D., Saponaro M., Sarcognato S., Guido M., Ferri N., Carrara M., De Martin S. (2020). Fucus vesiculosus and Ascophyllum nodosum Ameliorate Liver Function by Reducing Diet-Induced Steatosis in Rats. Mar. Drugs.

[5] Golomb B.A., Evans M.A. (2008). Statin Adverse Effects: A Review of the Literature and Evidence for a Mitochondrial Mechanism. Am. J. Cardiovasc. Drugs.

[6] Lankatillake C., Huynh T., Dias D.A. (2019). Understanding glycaemic control and current approaches for screening antidiabetic natural products from evidence-based medicinal plants. Plant Methods.

[7] Gómez-Zorita S., González-Arceo M., Trepiana J., Eseberri I., Fernández-Quintela A., Milton-Laskibar I., Aguirre L., González M., Portillo M.P. (2020). Anti-Obesity Effects of Macroalgae. Nutrients.

[8] Shannon E., Abu-Ghannam N. (2019). Seaweeds as nutraceuticals for health and nutrition. Phycologia.

[9] Iso H. (2011). Lifestyle and Cardiovascular Disease in Japan. J. Atheroscler. Thromb..

[10] Nanri A., Mizoue T., Shimazu T., Ishihara J., Takachi R., Noda M., Iso H., Sasazuki S., Sawada N., Tsugane S. (2017). Dietary patterns and all-cause, cancer, and cardiovascular disease mortality in Japanese men and women: The Japan public health center-based prospective study. PLoS ONE.

[11] Brown E.S., Allsopp P.J., Magee P.J., Gill C.I.R., Nitecki S., Strain C.R., McSorley E.M. (2014). Seaweed and human health. Nutr. Rev..

[12] Flórez-Fernández N., Domínguez H., Torres M.D. (2019). A green approach for alginate extraction from Sargassum muticum brown seaweed using ultrasound-assisted technique. Int. J. Biol. Macromol..

[13] Flórez-Fernández N., Torres M.D., González-Muñoz M.J., Domínguez H. (2019). Recovery of bioactive and gelling extracts from edible brown seaweed Laminaria ochroleuca by non-isothermal autohydrolysis. Food Chem..

[14] Flórez-Fernández N., Álvarez-Viñas M., Guerreiro F., Torres M.D., Grenha A., Domínguez H. (2020). Hydrothermal Processing of Laminaria ochroleuca for the Production of Crude Extracts Used to Formulate Polymeric Nanoparticles. Mar. Drugs.

[15] Guiry M.D. (2010). AlgaeBase.

[16] Rindi F., Soler-vila A., Maggs C.A., Hayes M. (2012). Taxonomy of Marine Macroalgae Used as Sources of Bioactive Compounds. Marine Bioactive Compounds.

[17] Murugan A.C., Karim M.R., Yusoff M.B.M., Tan S.H., Asras M.F.B.F., Rashid S.S. (2015). New insights into seaweed polyphenols on glucose homeostasis. Pharm. Biol..

[18] Wijesinghe W.A.J.P., Jeon Y.J. (2011). Biological activities and potential cosmeceutical applications of bioactive components from brown seaweeds: A review. Phytochem. Rev..

[19] Fernando I.P.S., Ryu B., Ahn G., Yeo I.K., Jeon Y.J. (2020). Therapeutic potential of algal natural products against metabolic syndrome: A review of recent developments. Trends Food Sci. Technol..

[20] Shaw J.E., Sicree R.A., Zimmet P.Z. (2010). Global estimates of the prevalence of diabetes for 2010 and 2030. Diabetes Res. Clin. Pract..

[21] Testa R., Bonfigli A.R., Prattichizzo F., La Sala L., De Nigris V., Ceriello A. (2017). The “Metabolic Memory” Theory and the Early Treatment of Hyperglycemia in Prevention of Diabetic Complications. Nutrients.

[22] Lackland D.T., Weber M.A. (2015). Global burden of cardiovascular disease and stroke: Hypertension at the core. Can. J. Cardiol..

[23] Lee Y.H., Yoon S.J., Kim A., Seo H., Ko S. (2016). Health Performance and Challenges in Korea: A Review of the Global Burden of Disease Study 2013. J. Korean Med. Sci..

[24] Yamori Y., Sagara M., Arai Y., Kobayashi H., Kishimoto K., Matsuno I., Mori H., Mori M. (2017). Soy and fish as features of the Japanese diet and cardiovascular disease risks. PLoS ONE.

[25] Maruyama K., Iso H., Date C., Kikuchi S., Watanabe Y., Wada Y., Inaba Y., Tamakoshi A., JACC Study Group (2013). Dietary patterns and risk of cardiovascular deaths among middle-aged Japanese: JACC Study. Nutr. Metab. Cardiovasc. Dis..

[26] Chu S.M., Shih W.T., Yang Y.H., Chen P.C., Chu Y.H. (2015). Use of traditional Chinese medicine in patients with hyperlipidemia: A population-based study in Taiwan. J. Ethnopharmacol..

[27] Yki-Järvinen H. (2014). Non-alcoholic fatty liver disease as a cause and a consequence of metabolic syndrome. Lancet Diabetes Endocrinol..

[28] Gabbia D., Roverso M., Guido M., Sacchi D., Scaffidi M., Carrara M., Orso G., Russo F.P., Floreani A., Bogialli S. (2019). Western Diet-Induced Metabolic Alterations Affect Circulating Markers of Liver Function before the Development of Steatosis. Nutrients.

[29] Heilbronn L.K., Noakes M., Clifton P.M. (2001). Energy restriction and weight loss on very-low-fat diets reduce C-reactive protein concentrations in obese, healthy women. Arterioscler. Thromb. Vasc. Biol..

[30] Stranges S., Trevisan M., Dorn J.M., Dmochowski J., Donahue R.P. (2005). Body fat distribution, liver enzymes, and risk of hypertension: Evidence from the Western New York Study. Hypertension.

[31] Deniaud-Bouët E., Hardouin K., Potin P., Kloareg B., Hervé C. (2017). A review about brown algal cell walls and fucose-containing sulfated polysaccharides: Cell wall context, biomedical properties and key research challenges. Carbohydr. Polym..

[32] Lorenzo J.M., Agregán R., Munekata P.E.S., Franco D., Carballo J., Şahin S., Lacomba R., Barba F.J. (2017). Proximate Composition and Nutritional Value of Three Macroalgae: Ascophyllum nodosum, Fucus vesiculosus and Bifurcaria bifurcata. Mar. Drugs.

[33] Gupta S., Abu-Ghannam N. (2011). Bioactive potential and possible health effects of edible brown seaweeds. Trends Food Sci. Technol..

[34] Catarino M.D., Silva A.M.S., Cardoso S.M. (2017). Fucaceae: A Source of Bioactive Phlorotannins. Int. J. Mol. Sci..

[35] Catarino M.D., Silva A.M.S., Cardoso S.M. (2018). Phycochemical Constituents and Biological Activities of Fucus spp.. Mar. Drugs.

[36] Afonso N.C., Catarino M.D., Silva A.M.S., Cardoso S.M. (2019). Brown Macroalgae as Valuable Food Ingredients. Antioxidants.

[37] Brownlee I.A., Allen A., Pearson J.P., Dettmar P.W., Havler M.E., Atherton M.R., Onsøyen E. (2005). Alginate as a Source of Dietary Fiber. Crit. Rev. Food Sci. Nutr..

[38] Pádua D., Rocha E., Gargiulo D., Ramos A.A. (2015). Bioactive compounds from brown seaweeds: Phloroglucinol, fucoxanthin and fucoidan as promising therapeutic agents against breast cancer. Phytochem. Lett..

[39] Usman A., Khalid S., Usman A., Hussain Z., Wang Y., Zia K.M., Zuber M., Ali M. (2017). Chapter 5—Algal Polysaccharides, Novel Application, and Outlook. Algae Based Polymers, Blends, and Composites.

[40] Susanto E., Fahmi A.S., Abe M., Hosokawa M., Miyashita K. (2016). Lipids, Fatty Acids, and Fucoxanthin Content from Temperate and Tropical Brown Seaweeds. Aquat. Procedia.

[41] Silva A.F.R., Abreu H., Silva A.M.S., Cardoso S.M. (2019). Effect of Oven-Drying on the Recovery of Valuable Compounds from Ulva rigida, Gracilaria sp. and Fucus vesiculosus. Mar. Drugs.

[42] Zaharudin N., Salmeán A.A., Dragsted L.O. (2018). Inhibitory effects of edible seaweeds, polyphenolics and alginates on the activities of porcine pancreatic α-amylase. Food Chem..

[43] Lee S.H., Yong-Li, Karadeniz F., Kim M.M., Kim S.K. (2009). α-Glucosidase and α-amylase inhibitory activities of phloroglucinal derivatives from edible marine brown alga, Ecklonia cava. J. Sci. Food Agric..

[44] Jung H.A., Jung H.J., Jeong H.Y., Kwon H.J., Ali M.Y., Choi J.S. (2014). Phlorotannins isolated from the edible brown alga Ecklonia stolonifera exert anti-adipogenic activity on 3T3-L1 adipocytes by downregulating C/EBPα and PPARγ. Fitoterapia.

[45] Lee S.H., Park M.H., Heo S.J., Kang S.M., Ko S.C., Han J.S., Jeon Y.J. (2010). Dieckol isolated from Ecklonia cava inhibits alpha-glucosidase and alpha-amylase in vitro and alleviates postprandial hyperglycemia in streptozotocin-induced diabetic mice. Food Chem. Toxicol..

[46] Choi J.S., Han Y.R., Byeon J.S., Choung S.Y., Sohn H.S., Jung H.A. (2015). Protective effect of fucosterol isolated from the edible brown algae, Ecklonia stolonifera and Eisenia bicyclis, on tert-butyl hydroperoxide- and tacrine-induced HepG2 cell injury. J. Pharm. Pharmacol..

[47] Karadeniz F., Ahn B.N., Kim J.A., Seo Y., Jang M.S., Nam K.H., Kim M., Lee S.H., Kong C.S. (2015). Phlorotannins suppress adipogenesis in pre-adipocytes while enhancing osteoblastogenesis in pre-osteoblasts. Arch. Pharm. Res..

[48] Kong C.S., Kim H., Seo Y. (2015). Edible Brown Alga Ecklonia cava Derived Phlorotannin-Induced Anti-Adipogenic Activity in Vitro. J. Food Biochem..

[49] Park S.R., Kim J.H., Jang H.D., Yang S.Y., Kim Y.H. (2018). Inhibitory activity of minor phlorotannins from Ecklonia cava on α-glucosidase. Food Chem..

[50] Oh S., Son M., Lee H.S., Kim H.S., Jeon Y.J., Byun K. (2018). Protective Effect of Pyrogallol-Phloroglucinol-6,6-Bieckol from Ecklonia cava on Monocyte-Associated Vascular Dysfunction. Mar. Drugs.

[51] Lee H.A., Lee J.H., Han J.S. (2017). A phlorotannin constituent of Ecklonia cava alleviates postprandial hyperglycemia in diabetic mice. Pharm. Biol..

[52] Lee H.A., Lee J.H., Han J.S. (2018). 2,7”-Phloroglucinol-6,6′-bieckol protects INS-1 cells against high glucose-induced apoptosis. Biomed. Pharmacother..

[53] Eom S.H., Lee M.S., Lee E.W., Kim Y.M., Kim T.H. (2013). Pancreatic lipase inhibitory activity of phlorotannins isolated from Eisenia bicyclis. Phytother. Res..

[54] Son M., Oh S., Lee H.S., Ryu B., Jiang Y., Jang J.T., Jeon Y.J., Byun K. (2019). Pyrogallol-Phloroglucinol-6,6′-Bieckol from Ecklonia cava Improved Blood Circulation in Diet-Induced Obese and Diet-Induced Hypertension Mouse Models. Mar. Drugs.

[55] Ko S.C., Kang M.C., Kang N., Kim H.S., Lee S.H., Ahn G., Jung W.K., Jeon Y.J. (2017). Effect of angiotensin I-converting enzyme (ACE) inhibition and nitric oxide (NO) production of 6,6′-bieckol, a marine algal polyphenol and its anti-hypertensive effect in spontaneously hypertensive rats. Process Biochem..

[56] Lopes G., Barbosa M., Andrade P.B., Valentão P. (2019). Phlorotannins from Fucales: Potential to control hyperglycemia and diabetes-related vascular complications. J. Appl. Phycol..

[57] Liu M., Hansen P.E., Lin X. (2011). Bromophenols in marine algae and their bioactivities. Mar. Drugs.

[58] Shi D.Y., Xu F., Li J., Guo S.J., Su H., Han L.J. (2008). PTP1B inhibitory activities of bromophenol derivatives from algae. Zhongguo Zhong Yao Za Zhi.

[59] Sunderland A.M., Dettmar P.W., Pearson J.P. (2000). Alginates inhibit pepsin activity in vitro; A justification for their use in gastro-oesophageal reflux disease (gord). Gastroenterology.

[60] Chater P.I., Wilcox M.D., Brownlee I.A., Pearson J.P. (2015). Alginate as a protease inhibitor in vitro and in a model gut system; selective inhibition of pepsin but not trypsin. Carbohydr. Polym..

[61] Ben Gara A., Ben Abdallah Kolsi R., Jardak N., Chaaben R., El-Feki A., Fki L., Belghith H., Belghith K. (2017). Inhibitory activities of Cystoseira crinita sulfated polysaccharide on key enzymes related to diabetes and hypertension: In vitro and animal study. Arch. Physiol. Biochem..

[62] Li X., Li J., Li Z., Sang Y., Niu Y., Zhang Q., Ding H., Yin S. (2016). Fucoidan from Undaria pinnatifida prevents vascular dysfunction through PI3K/Akt/eNOS-dependent mechanisms in the l-NAME-induced hypertensive rat model. Food Funct..

[63] Wang Y., Xing M., Cao Q., Ji A., Liang H., Song S. (2019). Biological Activities of Fucoidan and the Factors Mediating Its Therapeutic Effects: A Review of Recent Studies. Mar. Drugs.

[64] Sim S.Y., Shin Y.E., Kim H.K. (2019). Fucoidan from Undaria pinnatifida has anti-diabetic effects by stimulation of glucose uptake and reduction of basal lipolysis in 3T3-L1 adipocytes. Nutr. Res..

[65] Zheng Y., Liu T., Wang Z., Xu Y., Zhang Q., Luo D. (2018). Low molecular weight fucoidan attenuates liver injury via SIRT1/AMPK/PGC1α axis in db/db mice. Int. J. Biol. Macromol..

[66] Kadam S.U., Tiwari B.K., O’Donnell C.P. (2015). Extraction, structure and biofunctional activities of laminarin from brown algae. Int. J. Food Sci. Technol..

[67] Kadam S.U., O’Donnell C.P., Rai D.K., Hossain M.B., Burgess C.M., Walsh D., Tiwari B.K. (2015). Laminarin from Irish Brown Seaweeds Ascophyllum nodosum and Laminaria hyperborea: Ultrasound Assisted Extraction, Characterization and Bioactivity. Mar. Drugs.

[68] Choi J.I., Kim H.J., Kim J.H., Lee J.W. (2012). Enhanced Biological Activities of Laminarin Degraded by Gamma-Ray Irradiation. J. Food Biochem..

[69] Neyrinck A.M., Mouson A., Delzenne N.M. (2007). Dietary supplementation with laminarin, a fermentable marine beta (1-3) glucan, protects against hepatotoxicity induced by LPS in rat by modulating immune response in the hepatic tissue. Int. Immunopharmacol..

[70] Lee J.Y., Kim Y.J., Kim H.J., Kim Y.S., Park W. (2012). Immunostimulatory effect of laminarin on RAW 264.7 mouse macrophages. Molecules.

[71] Judé S., Roger S., Martel E., Besson P., Richard S., Bougnoux P., Champeroux P., Le Guennec J.Y. (2006). Dietary long-chain omega-3 fatty acids of marine origin: A comparison of their protective effects on coronary heart disease and breast cancers. Prog. Biophys. Mol. Biol..

[72] Jung H.A., Islam M.N., Lee C.M., Jeong H.O., Chung H.Y., Woo H.C., Choi J.S. (2012). Promising antidiabetic potential of fucoxanthin isolated from the edible brown algae Eisenia bicyclis and Undaria pinnatifida. Fish. Sci..

[73] Maeda H., Hosokawa M., Sashima T., Murakami-Funayama K., Miyashita K. (2009). Anti-obesity and anti-diabetic effects of fucoxanthin on diet-induced obesity conditions in a murine model. Mol. Med. Rep..

[74] Maeda H., Hosokawa M., Sashima T., Takahashi N., Kawada T., Miyashita K. (2006). Fucoxanthin and its metabolite, fucoxanthinol, suppress adipocyte differentiation in 3T3-L1 cells. Int. J. Mol. Med..

[75] Zhang Y., Xu W., Huang X., Zhao Y., Ren Q., Hong Z., Huang M., Xing X. (2018). Fucoxanthin ameliorates hyperglycemia, hyperlipidemia and insulin resistance in diabetic mice partially through IRS-1/PI3K/Akt and AMPK pathways. J. Funct. Foods.

[76] Peng J., Yuan J.P., Wu C.F., Wang J.H. (2011). Fucoxanthin, a marine carotenoid present in brown seaweeds and diatoms: Metabolism and bioactivities relevant to human health. Mar. Drugs.

[77] Jung H.A., Islam M.N., Lee C.M., Oh S.H., Lee S., Jung J.H., Choi J.S. (2013). Kinetics and molecular docking studies of an anti-diabetic complication inhibitor fucosterol from edible brown algae Eisenia bicyclis and Ecklonia stolonifera. Chem. Biol. Interact..

[78] Song Y., Oh G.H., Kim M.B., Hwang J.K. (2017). Fucosterol inhibits adipogenesis through the activation of AMPK and Wnt/β-catenin signaling pathways. Food Sci. Biotechnol..

[79] Jung H.A., Jung H.J., Jeong H.Y., Kwon H.J., Kim M.S., Choi J.S. (2014). Anti-adipogenic activity of the edible brown alga Ecklonia stolonifera and its constituent fucosterol in 3T3-L1 adipocytes. Arch. Pharm. Res..

[80] Lee J.H., Jung H.A., Kang M.J., Choi J.S., Kim G.D. (2017). Fucosterol, isolated from Ecklonia stolonifera, inhibits adipogenesis through modulation of FoxO1 pathway in 3T3-L1 adipocytes. J. Pharm. Pharmacol..

[81] He W.F., Yao L.G., Liu H.L., Guo Y.W. (2014). Thunberol, a new sterol from the Chinese brown alga Sargassum thunbergii. J. Asian Nat. Prod. Res..

[82] Conde E., Balboa E.M., Parada M., Falqué E., Domínguez H. (2013). 4-Algal proteins, peptides and amino acids. Functional Ingredients from Algae for Foods and Nutraceuticals.

[83] Kang M.C., Ding Y., Kim E.A., Choi Y.K., de Araujo T., Heo S.J., Lee S.H. (2017). Indole Derivatives Isolated from Brown Alga Sargassum thunbergii Inhibit Adipogenesis through AMPK Activation in 3T3-L1 Preadipocytes. Mar. Drugs.

[84] Rupérez P., Ahrazem O., Leal J.A. (2002). Potential antioxidant capacity of sulfated polysaccharides from the edible marine brown seaweed Fucus vesiculosus. J. Agric. Food Chem..

[85] Biancarosa I., Belghit I., Bruckner C.G., Liland N.S., Waagbø R., Amlund H., Heesch S., Lock E.J. (2018). Chemical characterization of 21 species of marine macroalgae common in Norwegian waters: Benefits of and limitations to their potential use in food and feed. J. Sci. Food Agric..

[86] Circuncisão A.R., Catarino M.D., Cardoso S.M., Silva A.M.S. (2018). Minerals from Macroalgae Origin: Health Benefits and Risks for Consumers. Mar. Drugs.

[87] MacArtain P., Gill C.I.R., Brooks M., Campbell R., Rowland I.R. (2007). Nutritional value of edible seaweeds. Nutr. Rev..

[88] Moreda-Piñeiro J., Alonso-Rodríguez E., López-Mahía P., Muniategui-Lorenzo S., Prada-Rodríguez D., Moreda-Piñeiro A., Bermejo-Barrera P. (2007). Development of a new sample pre-treatment procedure based on pressurized liquid extraction for the determination of metals in edible seaweed. Anal. Chim. Acta.

[89] Paiva L., Lima E., Neto A.I., Marcone M., Baptista J. (2016). Health-promoting ingredients from four selected Azorean macroalgae. Food Res. Int..

[90] Murray M., Dordevic A.L., Bonham M.P., Ryan L. (2018). Do marine algal polyphenols have antidiabetic, antihyperlipidemic or anti-inflammatory effects in humans? A systematic review. Crit. Rev. Food Sci. Nutr..

[91] Koivikko R., Loponen J., Honkanen T., Jormalainen V. (2005). Contents of soluble, cell-wall-bound and exuded phlorotannins in the brown alga Fucus vesiculosus, with implications on their ecological functions. J. Chem. Ecol..

[92] Lopes G., Andrade P.B., Valentão P. (2017). Phlorotannins: Towards New Pharmacological Interventions for Diabetes Mellitus Type 2. Molecules.

[93] Moon H.E., Islam N., Ahn B.R., Chowdhury S.S., Sohn H.S., Jung H.A., Choi J.S. (2011). Protein tyrosine phosphatase 1B and α-glucosidase inhibitory Phlorotannins from edible brown algae, Ecklonia stolonifera and Eisenia bicyclis. Biosci. Biotechnol. Biochem..

[94] Lee S.H., Jeon Y.J. (2013). Anti-diabetic effects of brown algae derived phlorotannins, marine polyphenols through diverse mechanisms. Fitoterapia.

[95] Gabbia D., Dall’Acqua S., Di Gangi I.M., Bogialli S., Caputi V., Albertoni L., Marsilio I., Paccagnella N., Carrara M., Giron M.C. (2017). The Phytocomplex from Fucus vesiculosus and Ascophyllum nodosum Controls Postprandial Plasma Glucose Levels: An In Vitro and In Vivo Study in a Mouse Model of NASH. Mar. Drugs.

[96] Roy M.C., Anguenot R., Fillion C., Beaulieu M., Bérubé J., Richard D. (2011). Effect of a commercially-available algal phlorotannins extract on digestive enzymes and carbohydrate absorption in vivo. Food Res. Int..

[97] Heo S.J., Hwang J.Y., Choi J.I., Han J.S., Kim H.J., Jeon Y.J. (2009). Diphlorethohydroxycarmalol isolated from Ishige okamurae, a brown algae, a potent alpha-glucosidase and alpha-amylase inhibitor, alleviates postprandial hyperglycemia in diabetic mice. Eur. J. Pharmacol..

[98] Kwon T.H., Wu Y.X., Kim J.S., Woo J.H., Park K.T., Kwon O.J., Seo H.J., Kim T., Park N.H. (2015). 6,6′-Bieckol inhibits adipocyte differentiation through downregulation of adipogenesis and lipogenesis in 3T3-L1 cells. J. Sci. Food Agric..

[99] Dong H., Dong S., Erik Hansen P., Stagos D., Lin X., Liu M. (2020). Progress of Bromophenols in Marine Algae from 2011 to 2020: Structure, Bioactivities, and Applications. Mar. Drugs.

[100] Demir Y., Taslimi P., Ozaslan M.S., Oztaskin N., Çetinkaya Y., Gulçin İ., Beydemir Ş., Goksu S. (2018). Antidiabetic potential: In vitro inhibition effects of bromophenol and diarylmethanones derivatives on metabolic enzymes. Arch. Pharm..

[101] Rodrigues D., Freitas A.C., Pereira L., Rocha-Santos T.A.P., Vasconcelos M.W., Roriz M., Rodríguez-Alcalá L.M., Gomes A.M.P., Duarte A.C. (2015). Chemical composition of red, brown and green macroalgae from Buarcos bay in Central West Coast of Portugal. Food Chem..

[102] Kim M.S., Kim J.Y., Choi W.H., Lee S.S. (2008). Effects of seaweed supplementation on blood glucose concentration, lipid profile, and antioxidant enzyme activities in patients with type 2 diabetes mellitus. Nutr. Res. Pract..

[103] Rupérez P., Saura-Calixto F. (2001). Dietary fibre and physicochemical properties of edible Spanish seaweeds. Eur. Food Res. Technol..

[104] Chater P.I., Wilcox M.D., Houghton D., Pearson J.P. (2015). The role of seaweed bioactives in the control of digestion: Implications for obesity treatments. Food Funct..

[105] Lange K.W., Hauser J., Nakamura Y., Kanaya S. (2015). Dietary seaweeds and obesity. Food Sci. Hum. Wellness.

[106] Paxman J.R., Richardson J.C., Dettmar P.W., Corfe B.M. (2008). Alginate reduces the increased uptake of cholesterol and glucose in overweight male subjects: A pilot study. Nutr. Res..

[107] Wilcox M.D., Brownlee I.A., Richardson J.C., Dettmar P.W., Pearson J.P. (2014). The modulation of pancreatic lipase activity by alginates. Food Chem..

[108] Ficko-Blean E., Hervé C., Michel G. (2015). Sweet and sour sugars from the sea: The biosynthesis and remodeling of sulfated cell wall polysaccharides from marine macroalgae. Perspect. Phycol..

[109] Pomin V.H. (2012). Fucanomics and galactanomics: Current status in drug discovery, mechanisms of action and role of the well-defined structures. Biochim. Biophys. Acta.

[110] Ruderman N.B., Carling D., Prentki M., Cacicedo J.M. (2013). AMPK, insulin resistance, and the metabolic syndrome. J. Clin. Investig..

[111] Zhou M., Ding Y., Cai L., Wang Y., Lin C., Shi Z. (2018). Low molecular weight fucoidan attenuates experimental abdominal aortic aneurysm through interfering the leukocyte-endothelial cells interaction. Mol. Med. Rep..

[112] Paradis M.E., Couture P., Lamarche B. (2011). A randomised crossover placebo-controlled trial investigating the effect of brown seaweed (*Ascophyllum nodosum* and *Fucus vesiculosus*) on postchallenge plasma glucose and insulin levels in men and women. Appl. Physiol. Nutr. Metab..

[113] Chawla A., Nguyen K.D., Goh Y.P.S. (2011). Macrophage-mediated inflammation in metabolic disease. Nat. Rev. Immunol..

[114] Holdt S.L., Kraan S. (2011). Bioactive compounds in seaweed: Functional food applications and legislation. J. Appl. Phycol..

[115] Bae M., Kim M.B., Park Y.K., Lee J.Y. (2020). Health benefits of fucoxanthin in the prevention of chronic diseases. Biochim. Biophys. Acta Mol. Cell Biol. Lipids.

[116] Kim M.B., Bae M., Hu S., Kang H., Park Y.K., Lee J.Y. (2019). Fucoxanthin exerts anti-fibrogenic effects in hepatic stellate cells. Biochem. Biophys. Res. Commun..

[117] Takatani N., Kono Y., Beppu F., Okamatsu-Ogura Y., Yamano Y., Miyashita K., Hosokawa M. (2020). Fucoxanthin inhibits hepatic oxidative stress, inflammation, and fibrosis in diet-induced nonalcoholic steatohepatitis model mice. Biochem. Biophys. Res. Commun..

[118] Lopes G., Sousa C., Bernardo J., Andrade P.B., Valentão P., Ferreres F., Mouga T. (2011). Sterol Profiles in 18 Macroalgae of the Portuguese Coast1. J. Phycol..

[119] Moghadasian M.H., Frohlich J.J. (1999). Effects of dietary phytosterols on cholesterol metabolism and atherosclerosis: Clinical and experimental evidence. Am. J. Med..

[120] Hannan M.A., Sohag A.M.A., Dash R., Haque M.N., Mohibbullah M., Oktaviani D.F., Hossain M.T., Choi H.J., Moon I.S. (2020). Phytosterols of marine algae—Insights into the potential health benefits and molecular pharmacology. Phytomedicine.

[121] Güven K.C., Percot A., Sezik E. (2010). Alkaloids in Marine Algae. Mar. Drugs.

[122] El Maghraby D.M., Fakhry E.M. (2015). Lipid content and fatty acid composition of Mediterranean macro-algae as dynamic factors for biodiesel production. Oceanologia.

[123] Patterson E., Wall R., Fitzgerald G.F., Ross R.P., Stanton C. (2012). Health implications of high dietary omega-6 polyunsaturated Fatty acids. J. Nutr. Metab..

[124] Husted K.S., Bouzinova E.V. (2016). The importance of n-6/n-3 fatty acids ratio in the major depressive disorder. Medicina.

[125] Logan A.C. (2003). Neurobehavioral aspects of omega-3 fatty acids: Possible mechanisms and therapeutic value in major depression. Altern. Med. Rev..

[126] Simopoulos A.P. (2016). An Increase in the Omega-6/Omega-3 Fatty Acid Ratio Increases the Risk for Obesity. Nutrients.

[127] Kanoh M., Inai K., Shinohara T., Tomimatsu H., Nakanishi T. (2017). Clinical implications of eicosapentaenoic acid/arachidonic acid ratio (EPA/AA) in adult patients with congenital heart disease. Heart Vessel..

[128] Gibson G.R., Hutkins R., Sanders M.E., Prescott S.L., Reimer R.A., Salminen S.J., Scott K., Stanton C., Swanson K.S., Cani P.D. (2017). Expert consensus document: The International Scientific Association for Probiotics and Prebiotics (ISAPP) consensus statement on the definition and scope of prebiotics. Nat. Rev. Gastroenterol. Hepatol..

[129] Huebbe P., Nikolai S., Schloesser A., Herebian D., Campbell G., Glüer C.C., Zeyner A., Demetrowitsch T., Schwarz K., Metges C.C. (2017). An extract from the Atlantic brown algae Saccorhiza polyschides counteracts diet-induced obesity in mice via a gut related multi-factorial mechanisms. Oncotarget.

[130] Phang S.M. (2010). Potential Products from Tropical Algae and Seaweeds, especially with Reference to Malaysia. Malays. J. Sci..

[131] Cofrades S., López-López I., Ruiz-Capillas C., Triki M., Jiménez-Colmenero F. (2011). Quality characteristics of low-salt restructured poultry with microbial transglutaminase and seaweed. Meat Sci..

[132] López-López I., Bastida S., Ruiz-Capillas C., Bravo L., Larrea M.T., Sánchez-Muniz F., Cofrades S., Jiménez-Colmenero F. (2009). Composition and antioxidant capacity of low-salt meat emulsion model systems containing edible seaweeds. Meat Sci..

[133] Schultz Moreira A.R., Benedí J., González-Torres L., Olivero-David R., Bastida S., Sánchez-Reus M.I., González-Muñoz M.J., Sánchez-Muniz F.J. (2011). Effects of diet enriched with restructured meats, containing Himanthalia elongata, on hypercholesterolaemic induction, CYP7A1 expression and antioxidant enzyme activity and expression in growing rats. Food Chem..

[134] Moreira A.S., González-Torres L., Olivero-David R., Bastida S., Benedi J., Sánchez-Muniz F.J. (2010). Wakame and Nori in restructured meats included in cholesterol-enriched diets affect the antioxidant enzyme gene expressions and activities in Wistar rats. Plant Foods Hum. Nutr..

[135] Schultz Moreira A.R., Benedi J., Bastida S., Sánchez-Reus I., Sánchez-Muniz F.J. (2013). Nori- and sea spaghetti- but not wakame-restructured pork decrease the hypercholesterolemic and liver proapototic short-term effects of high-dietary cholesterol consumption. Nutr. Hosp..

[136] Kim H.H., Lim H.S. (2014). Effects of Sea Tangle-added Patty on Postprandial Serum Lipid Profiles and Glucose in Borderline Hypercholesterolemic Adults. J. Korean Soc. Food Sci. Nutr..

[137] López-López I., Cofrades S., Jiménez-Colmenero F. (2009). Low-fat frankfurters enriched with n-3 PUFA and edible seaweed: Effects of olive oil and chilled storage on physicochemical, sensory and microbial characteristics. Meat Sci..

[138] López-López I., Cofrades S., Ruiz-Capillas C., Jiménez-Colmenero F. (2009). Design and nutritional properties of potential functional frankfurters based on lipid formulation, added seaweed and low salt content. Meat Sci..

[139] Sellimi S., Ksouda G., Benslima A., Nasri R., Rinaudo M., Nasri M., Hajji M. (2017). Enhancing colour and oxidative stabilities of reduced-nitrite turkey meat sausages during refrigerated storage using fucoxanthin purified from the Tunisian seaweed Cystoseira barbata. Food Chem. Toxicol..

[140] Rico D., Linaje A.A.d., Herrero A., Asensio-Vegas C., Miranda J., Martínez-Villaluenga C., Luis D.A.d., Martin-Diana A.B. (2018). Carob by-products and seaweeds for the development of functional bread. J. Food Process. Preserv..

[141] Hall A.C., Fairclough A.C., Mahadevan K., Paxman J.R. (2012). Ascophyllum nodosum enriched bread reduces subsequent energy intake with no effect on post-prandial glucose and cholesterol in healthy, overweight males. A pilot study. Appetite.

[142] Prabhasankar P., Ganesan P., Bhaskar N., Hirose A., Stephen N., Gowda L.R., Hosokawa M., Miyashita K. (2009). Edible Japanese seaweed, wakame (Undaria pinnatifida) as an ingredient in pasta: Chemical, functional and structural evaluation. Food Chem..

[143] Martínez-Villaluenga C., Peñas E., Rico D., Martin-Diana A.B., Portillo M.P., Macarulla M.T., de Luis D.A., Miranda J. (2018). Potential Usefulness of a Wakame/Carob Functional Snack for the Treatment of Several Aspects of Metabolic Syndrome: From In Vitro to In Vivo Studies. Mar. Drugs.

[144] Astorga-España M.S., Mansilla A., Ojeda J., Marambio J., Rosenfeld S., Mendez F., Rodriguez J.P., Ocaranza P. (2017). Nutritional properties of dishes prepared with sub-Antarctic macroalgae—An opportunity for healthy eating. J. Appl. Phycol..

[145] González-Torres L., Churruca I., Schultz Moreira A.R., Bastida S., Benedí J., Portillo M.P., Sánchez-Muniz F.J. (2012). Effects of restructured pork containing Himanthalia elongata on adipose tissue lipogenic and lipolytic enzyme expression of normo- and hypercholesterolemic rats. J. Nutr..

[146] Olivero-David R., Schultz-Moreira A., Vázquez-Velasco M., González-Torres L., Bastida S., Benedí J., Sanchez-Reus M.I., González-Muñoz M.J., Sánchez-Muniz F.J. (2011). Effects of Nori- and Wakame-enriched meats with or without supplementary cholesterol on arylesterase activity, lipaemia and lipoproteinaemia in growing Wistar rats. Br. J. Nutr..

[147] Schultz Moreira A.R., Olivero-David R., Vázquez-Velasco M., González-Torres L., Benedí J., Bastida S., Sánchez-Muniz F.J. (2014). Protective effects of sea spaghetti-enriched restructured pork against dietary cholesterol: Effects on arylesterase and lipoprotein profile and composition of growing rats. J. Med. Food.

[148] Schultz Moreira A.R., García-Fernández R.A., Bocanegra A., Méndez M.T., Bastida S., Benedí J., Sánchez-Reus M.I., Sánchez-Muniz F.J. (2013). Effects of seaweed-restructured pork diets enriched or not with cholesterol on rat cholesterolaemia and liver damage. Food Chem. Toxicol..

[149] Cox S., Abu-Ghannam N. (2013). Enhancement of the phytochemical and fibre content of beef patties with Himanthalia elongata seaweed. Int. J. Food Sci. Technol..

[150] Dolea D., Rizo A., Fuentes A., Barat J., Fernández-Segovia I. (2018). Effect of thyme and oregano essential oils on the shelf life of salmon and seaweed burgers. Food Sci. Technol. Int..

[151] Abu-Ghannam N., Shannon E. (2017). Seaweed Carotenoid, Fucoxanthin, as Functional Food. Microbial Functional Foods and Nutraceuticals.

[152] Honold P.J., Jacobsen C., Jónsdóttir R., Kristinsson H.G., Hermund D.B. (2016). Potential seaweed-based food ingredients to inhibit lipid oxidation in fish-oil-enriched mayonnaise. Eur. Food Res. Technol..

[153] Cherry P., Yadav S., Strain C.R., Allsopp P.J., McSorley E.M., Ross R.P., Stanton C. (2019). Prebiotics from Seaweeds: An Ocean of Opportunity?. Mar. Drugs.

[154] Quintieri L., Fantin M., Palatini P., De Martin S., Rosato A., Caruso M., Geroni C., Floreani M. (2008). In vitro hepatic conversion of the anticancer agent nemorubicin to its active metabolite PNU-159682 in mice, rats and dogs: A comparison with human liver microsomes. Biochem. Pharmacol..

[155] Floreani M., Gabbia D., Barbierato M., De Martin S., Palatini P. (2012). Differential inducing effect of benzo[a]pyrene on gene expression and enzyme activity of cytochromes P450 1A1 and 1A2 in Sprague-Dawley and Wistar rats. Drug Metab. Pharmacokinet..

[156] Corona G., Ji Y., Anegboonlap P., Hotchkiss S., Gill C., Yaqoob P., Spencer J.P.E., Rowland I. (2016). Gastrointestinal modifications and bioavailability of brown seaweed phlorotannins and effects on inflammatory markers. Br. J. Nutr..

[157] Baldrick F.R., McFadden K., Ibars M., Sung C., Moffatt T., Megarry K., Thomas K., Mitchell P., Wallace J.M.W., Pourshahidi L.K. (2018). Impact of a (poly)phenol-rich extract from the brown algae Ascophyllum nodosum on DNA damage and antioxidant activity in an overweight or obese population: A randomized controlled trial. Am. J. Clin. Nutr..

[158] Nagamine T., Nakazato K., Tomioka S., Iha M., Nakajima K. (2014). Intestinal Absorption of Fucoidan Extracted from the Brown Seaweed, Cladosiphon okamuranus. Mar. Drugs.

[159] Kadena K., Tomori M., Iha M., Nagamine T. (2018). Absorption Study of Mozuku Fucoidan in Japanese Volunteers. Mar. Drugs.

[160] Tokita Y., Hirayama M., Nakajima K., Tamaki K., Iha M., Nagamine T. (2017). Detection of Fucoidan in Urine after Oral Intake of Traditional Japanese Seaweed, *Okinawa mozuku* (*Cladosiphon okamuranus* Tokida). J. Nutr. Sci. Vitaminol..

[161] Yonekura L., Nagao A. (2009). Soluble fibers inhibit carotenoid micellization in vitro and uptake by Caco-2 cells. Biosci. Biotechnol. Biochem..

[162] Hashimoto T., Ozaki Y., Taminato M., Das S.K., Mizuno M., Yoshimura K., Maoka T., Kanazawa K. (2009). The distribution and accumulation of fucoxanthin and its metabolites after oral administration in mice. Br. J. Nutr..

[163] Yonekura L., Kobayashi M., Terasaki M., Nagao A. (2010). Keto-carotenoids are the major metabolites of dietary lutein and fucoxanthin in mouse tissues. J. Nutr..

[164] Viera I., Pérez-Gálvez A., Roca M. (2018). Bioaccessibility of Marine Carotenoids. Mar. Drugs.

[165] Xi M., Dragsted L.O. (2019). Biomarkers of seaweed intake. Genes Nutr..

[166] Song W., Wang Z., Zhang X., Li Y. (2018). Ethanol Extract from Ulva prolifera Prevents High-Fat Diet-Induced Insulin Resistance, Oxidative Stress, and Inflammation Response in Mice. Biomed Res. Int..

[167] Zhao C., Yang C., Chen M., Lv X., Liu B., Yi L., Cornara L., Wei M.C., Yang Y.C., Tundis R. (2018). Regulatory Efficacy of Brown Seaweed Lessonia nigrescens Extract on the Gene Expression Profile and Intestinal Microflora in Type 2 Diabetic Mice. Mol. Nutr. Food Res..

[168] Kellogg J., Grace M.H., Lila M.A. (2014). Phlorotannins from Alaskan seaweed inhibit carbolytic enzyme activity. Mar. Drugs.

[169] Sharifuddin Y., Chin Y.X., Lim P.E., Phang S.M. (2015). Potential Bioactive Compounds from Seaweed for Diabetes Management. Mar. Drugs.

[170] Haskell-Ramsay C.F., Jackson P.A., Dodd F.L., Forster J.S., Bérubé J., Levinton C., Kennedy D.O. (2018). Acute Post-Prandial Cognitive Effects of Brown Seaweed Extract in Humans. Nutrients.

[171] Derosa G., Cicero A.F.G., D’Angelo A., Maffioli P. (2019). Ascophyllum nodosum and Fucus vesiculosus on glycemic status and on endothelial damage markers in dysglicemic patients. Phytother. Res..

[172] De Martin S., Gabbia D., Carrara M., Ferri N. (2018). The brown algae fucus vesiculosus and ascophyllum nodosum reduce metabolic syndrome risk factors: A clinical study. Nat. Prod. Commun..

[173] Murray M., Dordevic A.L., Ryan L., Bonham M.P. (2018). The Impact of a Single Dose of a Polyphenol-Rich Seaweed Extract on Postprandial Glycaemic Control in Healthy Adults: A Randomised Cross-Over Trial. Nutrients.

[174] Lee S.H., Jeon Y.J. (2015). Efficacy and safety of a dieckol-rich extract (AG-dieckol) of brown algae, Ecklonia cava, in pre-diabetic individuals: A double-blind, randomized, placebo-controlled clinical trial. Food Funct..

[175] Shin H.C., Kim S.H., Park Y., Lee B.H., Hwang H.J. (2012). Effects of 12-week oral supplementation of Ecklonia cava polyphenols on anthropometric and blood lipid parameters in overweight Korean individuals: A double-blind randomized clinical trial. Phytother. Res..

[176] Choi E.K., Park S.H., Ha K.C., Noh S.O., Jung S.J., Chae H.J., Chae S.W., Park T.S. (2015). Clinical trial of the hypolipidemic effects of a brown alga Ecklonia cava extract in patients with hypercholesterolemia. Int. J. Pharmacol..

[177] Tanemura Y., Yamanaka-Okumura H., Sakuma M., Nii Y., Taketani Y., Takeda E. (2014). Effects of the intake of Undaria pinnatifida (Wakame) and its sporophylls (Mekabu) on postprandial glucose and insulin metabolism. J. Med. Investig..

[178] Hata Y., Nakajima K., Uchida J., Hidaka H., Nakano T. (2001). Clinical Effects of Brown Seaweed, Undaria pinnatifida (wakame), on Blood Pressure in Hypertensive Subjects. J. Clin. Biochem. Nutr..

[179] Teas J., Baldeón M.E., Chiriboga D.E., Davis J.R., Sarriés A.J., Braverman L.E. (2009). Could dietary seaweed reverse the metabolic syndrome?. Asia Pac. J. Clin. Nutr..

[180] Abidov M., Ramazanov Z., Seifulla R., Grachev S. (2010). The effects of Xanthigen in the weight management of obese premenopausal women with non-alcoholic fatty liver disease and normal liver fat. Diabetes Obes. Metab..

[181] Hitoe S., Shimoda H. (2017). Seaweed Fucoxanthin Supplementation Improves Obesity Parameters in Mild Obese Japanese Subjects. Funct. Foods Health Dis..

[182] Hernández-Corona D.M., Martínez-Abundis E., González-Ortiz M. (2014). Effect of fucoidan administration on insulin secretion and insulin resistance in overweight or obese adults. J. Med. Food.

[183] Kang Y.M., Lee B.J., Kim J.I., Nam B.H., Cha J.Y., Kim Y.M., Ahn C.B., Choi J.S., Choi I.S., Je J.Y. (2012). Antioxidant effects of fermented sea tangle (*Laminaria japonica*) by Lactobacillus brevis BJ20 in individuals with high level of γ-GT: A randomized, double-blind, and placebo-controlled clinical study. Food Chem. Toxicol..

[184] Mikami N., Hosokawa M., Miyashita K., Sohma H., Ito Y.M., Kokai Y. (2017). Reduction of HbA1c levels by fucoxanthin-enriched akamoku oil possibly involves the thrifty allele of uncoupling protein 1 (UCP1): A randomised controlled trial in normal-weight and obese Japanese adults. J. Nutr. Sci..

[185] Murray M., Dordevic A.L., Cox K.H.M., Scholey A., Ryan L., Bonham M.P. (2018). Study protocol for a double-blind randomised controlled trial investigating the impact of 12 weeks supplementation with a Fucus vesiculosus extract on cholesterol levels in adults with elevated fasting LDL cholesterol who are overweight or have obesity. BMJ Open.

[186] Choi J.S., Seo H.J., Lee Y.R., Kwon S.J., Moon S.H., Park S.M., Sohn J.H. (2014). Characteristics and in vitro Anti-diabetic Properties of the Korean Rice Wine, Makgeolli Fermented with Laminaria japonica. Prev. Nutr. Food Sci..

[187] O’Sullivan A.M., O’Callaghan Y.C., O’Grady M.N., Queguineur B., Hanniffy D., Troy D.J., Kerry J.P., O’Brien N.M. (2012). Assessment of the ability of seaweed extracts to protect against hydrogen peroxide and tert-butyl hydroperoxide induced cellular damage in Caco-2 cells. Food Chem..

[188] Colognesi M., Gabbia D., De Martin S. (2020). Depression and Cognitive Impairment-Extrahepatic Manifestations of NAFLD and NASH. Biomedicines.

[189] Wada K., Tsuji M., Nakamura K., Oba S., Nishizawa S., Yamamoto K., Watanabe K., Ando K., Nagata C. (2020). Effect of dietary nori (dried laver) on blood pressure in young Japanese children: An intervention study. J. Epidemiol..

[190] Torres M.D., Arufe S., Chenlo F., Moreira R. (2017). Coeliacs cannot live by gluten-free bread alone—Every once in awhile they need antioxidants. Int. J. Food Sci. Technol..

